# Efficacy of flavonoids-containing supplements on insulin resistance and associated metabolic risk factors in overweight and obese subjects: a systematic review and meta-analysis of 25 randomized controlled trials

**DOI:** 10.3389/fendo.2022.917692

**Published:** 2022-07-22

**Authors:** Jia Yao, Yuan Zhang, Jia Zhao, Xian-Zhe Wang, Yu-Ping Lin, Lu Sun, Qi-Yun Lu, Guan-Jie Fan

**Affiliations:** ^1^ School of Second Clinical Medicine, Guangzhou University of Chinese Medicine, Guangzhou, China; ^2^ Department of Endocrinology, The Second Affiliated Hospital of Guangzhou University of Chinese Medicine, Guangzhou, China; ^3^ Department of Endocrinology, Guangdong Provincial Hospital of Chinese Medicine, Guangzhou, China

**Keywords:** Flavonoids, insulin resistance, overweight, obesity, systematic review, meta-analysis

## Abstract

**Background:**

Obesity is becoming a global epidemic. Flavonoids, with anti-inflammatory and antioxidative functions, are proposed to treat insulin resistance (IR) in obese subjects. We aimed to evaluate the effectiveness and safety of flavonoids-containing supplements on IR and associated metabolic risk factors in overweight and obese participants.

**Methods:**

Randomized controlled trials (RCTs) involving flavonoids-containing supplements used to treat overweight and obese subjects with results of IR, other associated metabolic risk factors, and adverse effects published were retrieved from 5 electronic databases from the year of inception to January 2, 2022.

**Results:**

Twenty-five RCTs (n = 1950) were included. Pooled results demonstrated that HOMA-IR in the group receiving flavonoids-containing supplements significantly decreased versus the control group (WMD = -0.132, 95% CI: -0.236 to -0.027, *p* = 0.013). Subgroup analyses showed that HOMA-IR in the subgroup receiving flavonoid-containing mixtures significantly decreased (WMD = -0.25, 95% CI: -0.43 to -0.06, *p* = 0.008), whereas such result was not found in the singly-used flavonoids subgroup (WMD = -0.08, 95% CI: -0.20 to 0.05, *p* = 0.240). In addition, QUICKI in the experimental group had an increasing trend compared to that in the control group (WMD = 0.01, 95% CI: -0.00 to 0.02, *p* = 0.065). For secondary outcomes, FBG, FBI, TC, TG, SBP, weight, BMI, and WHR in the group receiving flavonoids-containing supplements dropped significantly compared to those in the controls (WMD = -0.05, 95% CI: -0.08 to -0.02, *p* = 0.002; WMD = -0.58, 95% CI: -1.04 to -0.12, *p* = 0.014; WMD = -0.04, 95% CI: -0.06 to -0.03, *p* < 0.001; WMD = -0.04, 95% CI: -0.05 to -0.03, *p* < 0.001; WMD = -2.01, 95% CI: -3.17 to -0.86, *p* = 0.001; WMD = -0.29, 95% CI: -0.49 to -0.09, *p* = 0.004; WMD = -0.10 95% CI: -0.17 to -0.04, *p* = 0.003; WMD = -0.10, 95% CI: -0.01 to -0.00, *p* = 0.015; respectively). Adverse reactions did not differ between the group receiving flavonoids-containing supplements and the control group (RR = 0.97, 95% CI: 0.62 to 1.52, *p* = 0.905).

**Conclusion:**

This study showed that flavonoids-containing supplements may be efficacious and safe in improving IR and associated metabolic risk factors in overweight and obese participants. Nevertheless, doubt over the findings remains because limited RCTs per type of flavonoids-containing supplement were investigated, and many of the RCTs had a small sample size. Therefore, the findings must be validated in future research.

**Systematic Review Registration:**

https://inplasy.com/inplasy-2022-2-0011/, identifier INPLASY202220011.

## 1 Introduction

Obesity is becoming a global epidemic, which perplexes people worldwide. Obesity has nearly tripled worldwide since the 1970s (1). In 2016, more than 1.9 billion adults (39% of the global adult population) were overweight, of which over 650 million were obese (2). Obesity is one of the greatest health hazards, posing a significant burden on affected individuals, healthcare systems, and the entire society (3). Obesity is a major risk factor for the onset and progression of insulin resistance (IR). Obese and overweight subjects commonly develop IR, which is caused in part by the development of lipotoxicity in non-adipose tissues (4). Furthermore, obesity escalates the pathogenesis of various metabolic diseases (such as metabolic syndrome, type 2 diabetes, and cardiovascular disease) through the stimulation of IR.

Current treatment strategies for IR and obesity are primarily focused on lifestyle, pharmacologic, or surgical interventions. However, these interventions demonstrated significant interindividual variability in response, which further requires additional strategies to optimize the treatment of obesity and its associated metabolic disorders as mentioned above (5). Clinicians are currently treating obese and overweight patients with natural compounds isolated from the plant kingdom, and flavonoids appear to be a promising option. Flavonoids, a type of dietary polyphenol, are found in herbs, plant-based food, and beverages. Flavonoids are classified into flavonols, flavones, flavanones, flavan-3-ols, anthocyanins, and isoflavones based on their chemical structures (6–8). Flavonoids are proposed to treat IR in obese subjects because they have a range of physiologic effects including anti-inflammatory and antioxidative functions (9), making them a current focus in treating human metabolic diseases. To date, some randomized controlled trials (RCTs) were conducted concerning the efficacy of flavonoid-containing supplements in treating overweight and obese subjects. However, these RCTs showed inconsistent results, and the evidence remains decentralized. Therefore, this study made a systematic review and meta-analysis of the available evidence on the efficacy of flavonoid-containing supplements on IR and associated metabolic risk factors in overweight and obese participants.

## 2 Materials and methods

The current systematic review and meta-analysis were conducted following PRISMA 2020 statement (10). INPLASY registration number is INPLASY202220011, which is available from https://inplasy.com/inplasy-2022-2-0011/

### 2.1 Literature searches

From the inception to January 2, 2022, databases such as Pubmed, Embase, Web of Science, and the Cochrane Library were searched. Unpublished trials were also searched on the ClinicalTrials.gov registry, and the authors were contacted for further information as necessary. Search keywords, developed with the help of an expert medical librarian, included “flavonoid“, “flavonol”, “flavone”, “flavanone”, “flavan-3-ols”, “anthocyanidin”, “isoflavone”, “insulin resistance”, “overweight”, “obesity”, and “trial”. Only human subjects and RCTs were included in the search approach. The Supplementary Appendix listed the search syntaxes utilized. References for the included studies were also gathered to find any additional research that was not found during the original electronic search. There were no restrictions on language, publishing year, or type of publication.

### 2.2 Inclusion and exclusion criteria

The inclusion criteria were as follows :(1) RCTs with any length of follow-up and sample size; (2) subjects who are overweight or obese, regardless of gender, age, or ethnicity; (3) flavonoid-containing supplements were used as interventions in the experimental group; (4) primary measures: homeostasis model assessment of insulin resistance (HOMA-IR) and quantitative insulin sensitivity check index (QUICKI); secondary outcomes: fasting blood glucose (FBG), fasting blood insulin (FBI), blood lipids, blood pressure, weight, body mass index (BMI), waist circumference (WC), waist-to-hip ratio (WHR), and adverse effects.

The exclusion criteria were as follows: (1) RCTs in which outcome measures were inappropriate or relevant information could not be acquired from the authors; (2) non-RCTs, animal experiments, or reviews; (3) published literature that had already been reported.

### 2.3 Data extraction

After training and calibrating exercises, a pair of reviewers (J.Y. and Y.Z.) extracted data individually for each qualified trial using a predefined, pilot-tested data extraction form. They gathered data on trial features (author, year of publication, design, and sample size), patient characteristics (age, sex, and BMI), interventions in the experimental and control groups, dosage, route of administration, duration, study population, and desired outcomes. The differences of opinion were solved through negotiation and, when the help of a third party was enlisted if necessary (J.Z.). When the important details of a study were missing, we emailed the associated author(s) and searched the ClinicalTrials.gov database for more information.

### 2.4 Quality assessment

Reviewers assessed the risk of bias for each eligible trial using a revised Cochrane tool (RoB 2.0) (11). Bias risks were classified into four levels: low risk, some concerns—probably low risk, some concerns—probably high risk, and high risk in the following areas: randomization process, deviations from intended interventions, missing outcome data, measurement of the outcome, and selection of the reported result. The evaluation consisted of a series of signaling questions within each area; a judgment about the danger of bias was made by an algorithm that maps responses to signaling questions to the suggested judgment. If a study was judged to be of low risk of bias in all areas, we graded it as low risk of bias overall. If a trial was considered to have some concerns of bias in at least one area but did not show a high risk of bias in any area, we graded it as having some concerns of bias overall. If a trial was deemed to have a high risk of bias in at least one area or have some concerns of bias in many areas that significantly reduced confidence in the result, we graded it as a high risk of bias overall. When disputes could not be addressed *via* negotiation, the reviewers turned to a third party for settlement.

### 2.5 Statistical analysis

For statistical analysis, Stata (version 16.0, StataCorp LLC) was utilized. After standardizing the units, we generated the weighted mean difference (WMD) with a 95% confidence interval (CI) for continuous data. We estimated the relative risk (RR) with 95% CIs for dichotomous data. The mean differences and standard deviations of the group receiving flavonoid-containing supplements and the control group were extracted to calculate the effect size (12). The χ2-based Cochran Q statistic and the I^2^ statistic were used to assess heterogeneity. When I^2^ < 50%, a fixed-effects model was employed to pool the estimates from different trials. After clinical heterogeneity between trials was removed, the random-effects model was utilized when I^2^ >= 50%. Wherever possible, quantitative data were pooled for meta-analysis. Where pooling was not available, the findings were presented narratively. To investigate the potential sources of heterogeneity, subgroup analyses and sensitivity analyses were performed. Subgroup analyses were carried out depending on treatments (the use of singly-used flavonoids or flavonoid-containing mixtures), flavonoid subclasses, duration, and route of administration. The leave-one-out method was used for sensitivity analyses to assess the impact of each research on the overall effect size. The funnel plot approach was used to investigate publication bias. To quantify the publication bias, the Egger’s test and Begg’s test were used. The trim and fill approach was also used to rectify the funnel asymmetry induced by publication bias. A statistically significant difference was established by *p* < 0.05.

## 3 Results

### 3.1 Search results

**Figure 1:**
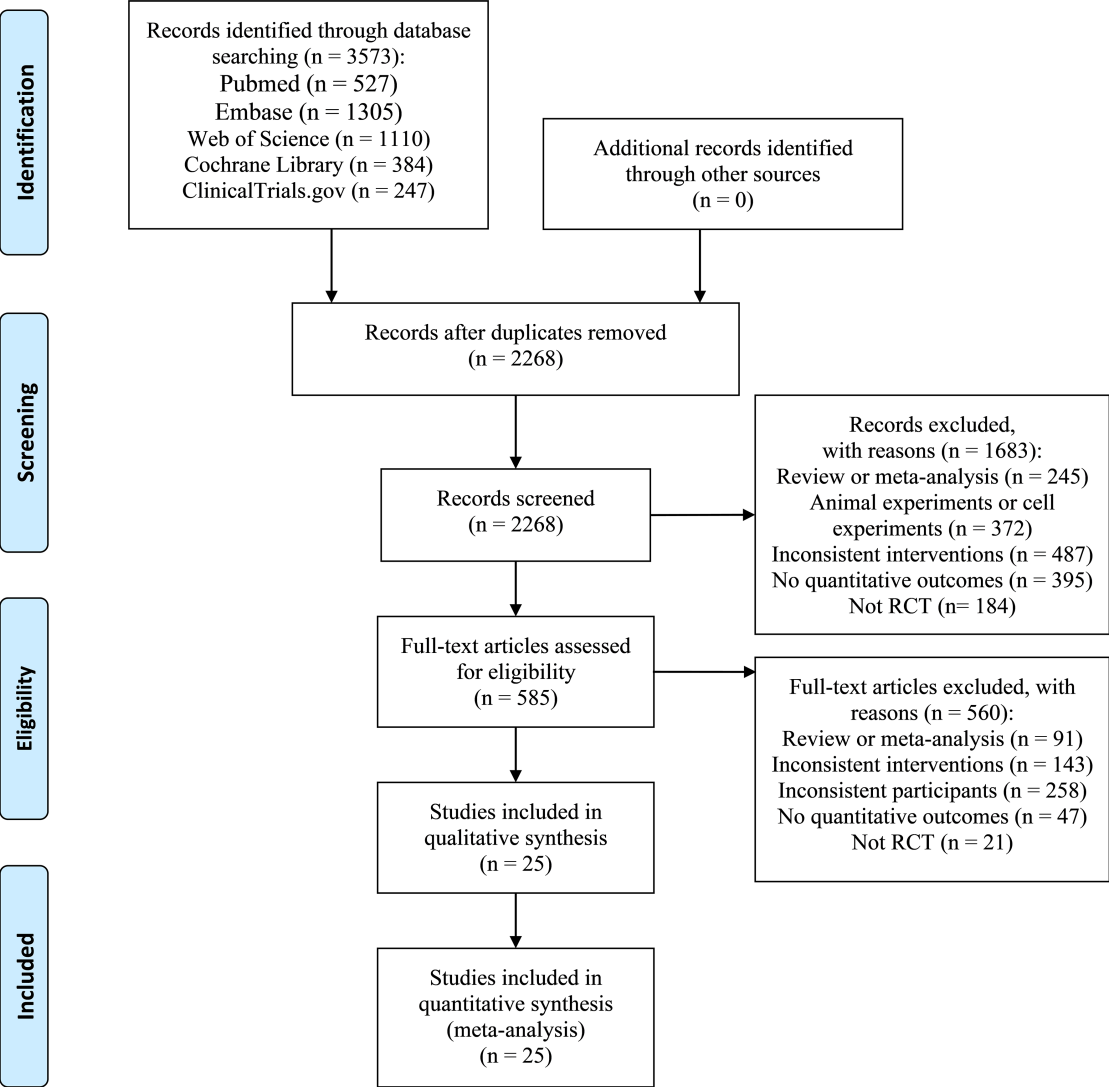
Flow diagram of study selection.

We retrieved 3,573 citations with 1,305 duplicates (**Figure 1**). After a preliminary screening of the titles and abstracts, we selected 585 studies for further full-text review. We then removed 560 studies, of which 47 did not supply quantitative outcome indicators, 21 were non-RCTs, 91 were reviews or meta-analyses, and the rest studies had unwanted interventions or subjects. We corresponded with the authors of studies without specific data on outcomes by e-mail to obtain relevant information. Unfortunately, we did not obtain replies until the completion of this writing. Ultimately, 25 RCTs (13–37) were included.

### 3.2 Study characteristics

**Table 1** presented the baseline characteristics of the RCTs included in the present study. Twenty-five trials (13–37) comprising 1950 people were examined (1000 in the experimental group and 950 in the control group). Flavonoids-containing supplements received by the experimental group included isoflavone, Glavonoid™, epigallocatechin gallate (EGCG), quercetin, bergamot extract, flavanol cocoa, genistein, green tea extract, (-)-epicatechin, tart cherry juice, the co-formulation of EGCG and resveratrol, polyphenol, the co-formulation of N-oleyl-phosphatidylethanolamine and EGCG, cynara, hesperidin 2S, the co-formulation of catechin and EGCG, cocoa/chocolate, and trans-resveratrol-hesperetin co-formulation. In 16 RCTs, singly-used flavonoids were used for interventions. In the other 9 RCTs, flavonoid-containing mixtures were used for interventions. Principal subclasses of flavonoids in the treatment group were isoflavones in 5 RCTs, flavan-3-ols in 12 RCTs, flavonols in 2 RCTs, flavanones in 3 RCTs, and multiple subclasses in 3 RCTs. The routes of administration included taking capsules and drinking beverages. The sample size varied from 22 to 237 individuals, the duration was from 7 to 12 months, and the average age was from 25.7 to 60.9 years. Thirteen RCTs (15, 17, 21, 23, 24, 28–32, 34, 36, 37) included overweight and obese subjects, 4 RCTs (13, 14, 19, 22) included overweight and obese postmenopausal women, 2 RCTs (16, 18) included overweight and obese subjects with hypertension, 1 RCT (20) included overweight dyslipidemic subjects, 1 RCT (25) included obese subjects with type 2 diabetes, 1 study (26) included overweight and obese women with polycystic ovary syndrome, 1 RCT (27) included overweight and obese subjects with metabolic syndrome, 1 study (33) included overweight and obese subjects with impaired fasting glycemia, and 1 RCT (35) included overweight breast cancer survivors.

**Table 1:** Baseline characteristics of the included trials.

Author,year	Group	Sample size	Intervention	Dosage and composition	Singly-used flavonoids or mixtures	Principal subclasses of flavonoids	Route of Administr-ation	Duration	Mean age (year)	Sex(M/F)	BMI (kg/m^2^)	Study Population	IR measure
Aubertin-leheudre, 2007 (13)	T	25	Isoflavone	Four capsules daily; each capsule contained 17.5 mg of isoflavones	Singly-used flavonoids	Isoflavones	Taking capsules	12 m	57.0 ± 5.0	0/25	30.0 ± 5.0	Obese postmenopausal women	QUICKI
	C	25	Placebo	A matched placebo					58.0 ± 5.0	0/25	30.0 ± 2.0		
Aubertin-leheudre, 2008 (14)	T	25	Isoflavone	Four capsules daily; each capsule contained 17.5 mg of isoflavones	Singly-used flavonoids	Isoflavones	Taking capsules	6 m	57.1 ± 5.6	0/25	31.2 ± 4.5	Obese postmenopausal women	HOMA-IR
	C	25	Placebo						57.7 ± 5.2	0/25	32.8 ± 4.8		
Bell, 2011 (15)	T	11	Glavonoid™	300 mg/day (taken in 3 capsules with the evening meal); Glavonoid™ is standardized to 30% licorice glabra polyphenol and 3% glabridin	Mixtures	Isoflavones	Taking capsules	8 w	28.4 ± 2.8	/	29.4 ± 1.3	Overweight and grade I-II obese subjects	HOMA-IR
	C	11	Placebo						25.7 ± 1.8	/	30.2 ± 1.2		
Bogdanski, 2012 (16)	T	28	EGCG	One capsule with their morning meal; the capsules contained 379 mg of green tea extract (including 208 mg of EGCG)	Singly-used flavonoids	Flavan-3-ols	Taking capsules	3 m	49.2 ± 8.8	13/15	32.5 ± 3.3	Obese hypertensive subjects	HOMA-IR
	C	28	Placebo	One capsule of pure microcrystalline cellulose					51.5 ± 7.4	15/13	33.9 ± 2.3		
Brown, 2009 (17)	T	46	EGCG	400 mg bid daily	Singly-used flavonoids	Flavan-3-ols	Taking capsules	8 w	52.2 ± 6.4	46/0	31.2 ± 2.8	Overweight and obese male subjects	HOMA-IR
	C	42	Placebo	A matched placebo					50.6 ± 6.5	42/0	31.0 ± 2.5		
Brüll, 2017 (18)	T	68	Quercetin	Three capsules per day (162 mg daily), one capsule with each principal meal	Singly-used flavonoids	Flavonols	Taking capsules	18 w	47.4 ± 10.5	34/34	31.1 ± 3.4	Overweight−to−obese patients with (pre−) hypertension	HOMA-IR
	C	68	Placebo	A matched placebo					47.4 ± 10.5	34/34	31.1 ± 3.4		
Choquette, 2011 (19)	T	23	Isoflavones	The 70 mg daily dose of isoflavones contained 44 mg of daidzein, 16 mg of glycitein and 10 mg of genistein	Singly-used flavonoids	Isoflavones	Taking capsules	6 m	58.0 ± 5.0	0/23	29.2 ± 2.4	Overweight-to-obese postmenopausal women	HOMA-IR
	C	22	Placebo	The placebo capsules contained cellulose only					59.0 ± 6.0	0/22	31.0 ± 2.9		
Cicero, 2019 (20)	T	30	Low-dose bergamot extract	Two pills at bedtime daily. The high‐dose group was given two boxes containing active treatment (bergamot extract (120mg flavonoids/pill)), and the low‐dose group was given one box containing active treatment and another one containing placebo	Mixtures	Multiple subclasses	Taking pills	24 w	43.0 ± 4.0	17/13	26.8 ± 1.7	Overweight dyslipidemic subjects	HOMA-IR
	T	30	High-dose bergamot extract						45.0 ± 4.0	14/16	26.5 ± 1.9		
	C	30	Placebo	Two boxes both containing placebo pills					44.0 ± 2.0	14/16	27.0 ± 1.8		
Davison, 2008 (21)	T	12	High dose-flavanol	902 mg flavanols daily	Singly-used flavonoids	Flavan-3-ols	Drinking beverage	12 w	45.3 ± 4.4	4/8	32.8 ± 1.1	Overweight and obese subjects	HOMA-IR
	C	11	Low dose-flavanol	36 mg flavanols daily					44.4 ± 4.4	3/8	34.5 ± 1.8		
Dostal, 2015 (22)	T	117	EGCG	Four green tea extract capsules containing 1315 ± 116 mg total catechins per day (843 ± 44 mg as EGCG)	Singly-used flavonoids	Flavan-3-ols	Taking capsules	12 m	60.9 ± 0.5	0/117	28.5 ± 0.3	Overweight and obese postmenopausal women	HOMA-IR
	C	120	Placebo	A matched placebo					60.6 ± 0.5	0/120	27.9 ± 0.3		
Guevara-Cruz, 2020 (23)	T	22	Genistein	50 mg/day	Singly-used flavonoids	Isoflavones	Taking capsules	2 m	42.6 ± 1.9	/	34.6 ± 0.9	Obese subjects	HOMA-IR
	C	23	Placebo	A matched placebo					43.0 ± 2.28	/	34.5 ± 1.0		
Hsu, 2008 (24)	T	41	Green tea extract	One capsule (400 mg) three times daily	Singly-used flavonoids	Flavan-3-ols	Taking capsules	12 w	43.0 ± 11.1	0/41	31.2 ± 3.5	Obese women	HOMA-IR
	C	37	Placebo	400 mg cellulose three times daily					43.9 ± 12.6	0/37	30.5 ± 4.6		
Hsu, 2011 (25)	T	35	Green tea extract	One capsule 30 minutes after meals three times daily; capsules contained 500 mg decaffeinated green tea extract	Singly-used flavonoids	Flavan-3-ols	Taking capsules	16 w	50.5 ± 9.2	12/23	30.3 ± 4.3	Obese type 2 diabetics	HOMA-IR
	C	33	Placebo	Pure microcrystalline cellulose					52.2 ± 9.1	12/21	29.2 ± 3.6		
Khorshidi, 2018 (26)	T	39	Quercetin	1,000 mg daily	Singly-used flavonoids	Flavonols	Taking capsules	12 w	29.5 ± 4.2	0/39	29.6 ± 3.7	Overweight or obese women with polycystic ovary syndrome	HOMA-IR
	C	39	Placebo	A matched placebo					30 ± 5.5	0/39	28.6 ± 4.1		
Kirch, 2018 (27)	T	47	(–)-epicatechin	25 mg daily	Singly-used flavonoids	Flavan-3-ols	Taking capsules	7 w	Males: 36.0 ± 12.0; Females: 35.0 ± 16.0	25/22	Males: 34.3 ± 6.2; Females: 31.2 ± 4.8	Overweight or obese subjects with metabolic syndrome	HOMA-IR
	C	47	Placebo	A matched placebo					Males: 36.0 ± 12.0; Females: 35.0 ± 16.0	25/22	Males: 34.3 ± 6.2; Females: 31.2 ± 4.8		
Martin, 2019 (28)	T	26	Tart cherry juice	240 mL daily. Tart cherry juice contained 65 mg anthocyanins/L (15.6 mg/240 mL) and 33.6 g total phenolics/L (993.6 mg/240 mL)	Mixtures	Multiple subclasses	Drinking beverage	12 w	41.0 ± 11.0	8/18	31.3 ± 6.0	Overweight and obese adults	HOMA-IR; QUICKI
	C	26	Placebo	240 mL/day; no concentrations of anthocyanins or phenolics					41.0 ± 11.0	8/18	31.3 ± 6.0		
Mielgo-Ayuso, 2013 (29)	T	43	EGCG	300 mg daily	Singly-used flavonoids	Flavan-3-ols	Taking capsules	12 w	19-49	0/43	33.7 ± 2.6	Obese women	HOMA-IR
	C	40	Placebo	A matched placebo						0/40	34.3 ± 3.0		
Most, 2016 (30)	T	18	EGCG and resveratrol	EGCG 282 mg daily and resveratrol 80 mg daily	Mixtures	Flavan-3-ols	Taking capsules	12 w	36.1 ± 2.2	18/20	29.9 ± 0.6	Overweight and obese subjects	HOMA-IR
	C	20	Placebo	A matched placebo					38.7 ± 2.2		29.5 ± 0.7		
Rangel-Huerta, 2015 (31)	T	100	High polyphenol concentration	A daily dose of 582.5 mg hesperidin, 125 mg narirutin, and 34 mg didymin	Mixtures	Flavanones	Drinking beverage	31 w	/	/	33.2 ± 0.5	Obese and overweight adults	HOMA-IR
	C	100	Normal polyphenol concentration	237 mg hesperidin, 45 mg narirutin, and 17 mg didymin daily					/	/	33.1 ± 0.6		
Rondanelli, 2009 (32)	T	71	N-oleyl-phosphatidylethanolamine and EGCG	One capsule twice daily, 85 mg N-oleyl-phosphatidylethanolamine and 50 mg EGCG per capsule	Mixtures	Flavan-3-ols	Taking capsules	2 m	38.0 ± 10.0	18/53	/	Overweight subjects	HOMA-IR; QUICKI
	C	67	Placebo	A matched placebo					41.0 ± 11.0	14/53	/		
Rondanelli, 2020 (33)	T	27	Cynara	500 mg bid daily. Tablets containing 500 mg of artichoke extract (triple standardized to contain caffeoylquinic acids ≥ 5.0%; flavonoids ≥ 1.5%; cynaropicrin ≥ 1.0%)	Mixtures	Multiple subclasses	Taking capsules	8 w	51.4 ± 6.6	28/26	29.0 ± 3.6	Overweight and obese with impaired fasting glycemia	HOMA-IR
	C	27	Placebo	A matched placebo					51.6 ± 6.0		29.7 ± 2.5		
Salden, 2016 (34)	T	34	Hesperidin 2S	Two capsules each morning before the consumption of breakfast; each of capsules contained 250 mg hesperidin 2S	Singly-used flavonoids	Flavanones	Taking capsules	6 w	54.0 ± 15.0	17/17	28.2 ± 2.2	Healthy overweight subjects	QUICKI
	C	34	Placebo	A matched placebo					53.0 ± 14.0	12/22	29.7 ± 2.8		
Stendell-Hollis, 2010 (35)	T	23	Catechin and EGCG	960 mL of decaffeinated green tea daily. The green tea bags comprised between 550–700 mg tea solids, providing an average catechin dose of 58.91 mg bag and 32.21 mg EGCG per bag	Singly-used flavonoids	Flavan-3-ols	Drinking beverage	6 m	56.6 ± 8.1	0/23	31.0 ± 4.3	Overweight breast cancer survivors	HOMA-IR
	C	16	Placebo	960 mL daily. The placebo tea was specifically manufactured for use in tea intervention trials of this nature and contained no EGCG					57.8 ± 8.5	0/16	28.7 ± 3.8		
West, 2014 (36)	T	30	Cocoa/chocolate	37 g daily of dark chocolate and a sugar-free cocoa beverage (total cocoa = 22 g/d, total flavanols = 814 mg daily)	Mixtures	Flavan-3-ols	Drinking beverage	10 w	51.7 ± 1.2	15/15	27.8 ± 0.6	Overweight and moderately obese subjects	HOMA-IR
	C	30	Colour-matched controls	A low-flavanol chocolate bar and a cocoa-free beverage with no added sugar (total flavanols = 3 mg daily)					51.7 ± 1.2	15/15	27.8 ± 0.6		
Xue, 2016 (37)	T	29	Trans-resveratrol-hesperetin co-formulation	One capsule daily contained trans-resveratrol (90 mg) and hesperetin (120 mg)	Mixtures	Flavanones	Taking capsules	22 w	45.0 ± 13.0	8/21	30.0 ± 3.8	Overweight and obese subjects	HOMA-IR
	C	29	Placebo	One capsule daily with starch in place of bioactives in hard gelatin capsules					45.0 ± 13.0	8/21	30.0 ± 3.8		

M/F, male/female; BMI, body mass index; IR, insulin resistance; T, treatment group; C, control group; d, day; m, month; w, week. EGCG, epigallocatechin-3-gallate; QUICKI, quantitative insulin sensitivity check index; HOMA-IR, homeostasis model assessment of insulin resistance.

### 3.3 Quality assessment

**Figure 2:**
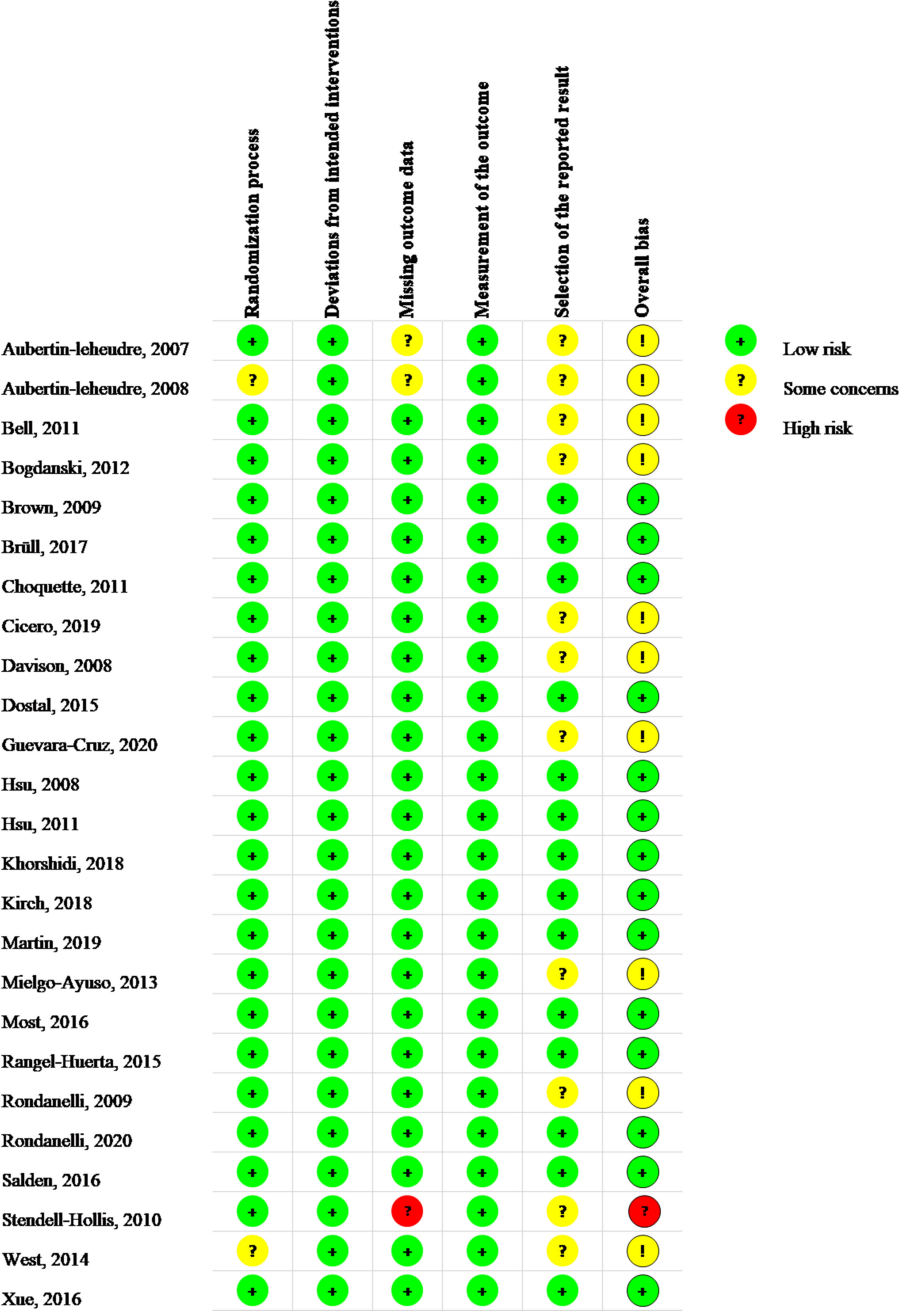
Risk of bias assessment in the included studies.

**Figure 2** illustrated the data of the risk of bias graded for the 25 RCTs that were included (13–37). Two trials (14, 36) (8%) had some concerns about the randomization procedure due to the lack of details about the concealment method and baseline data; the remaining 23 (92%) RCTs were graded as low risk. Low risk was assigned to the 25 trials based on deviations from the intended interventions. In terms of missing outcome data, one (4%) trial (35) was graded as high risk for its missing outcome data were more than 5%, which may have a detrimental influence on the intervention’s estimated effect. Two RCTs (8%) (13, 14) were flagged as having missing outcome data due to adverse events, but the missing data did not differ between the treatment and control groups. In the 25 RCTs, the measurement bias of the outcome was graded as low risk (100%). Regarding the selection bias of the reported results, for those without protocols or registrations, 11 (44%) RCTs (13–16, 20–23, 29, 32, 35, 36) were graded as having some concerns; the remaining 14 (56%) RCTs were graded as low risk. In terms of the overall risk of bias, 14 (56%) RCTs were graded as low risk, 10 (40%) RCTs were rated as having some issues, and one (4%) RCT was rated as high risk.

### 3.4 Pooled results

#### 3.4.1 Primary outcomes

(1) HOMA-IR

**Figure 3:**
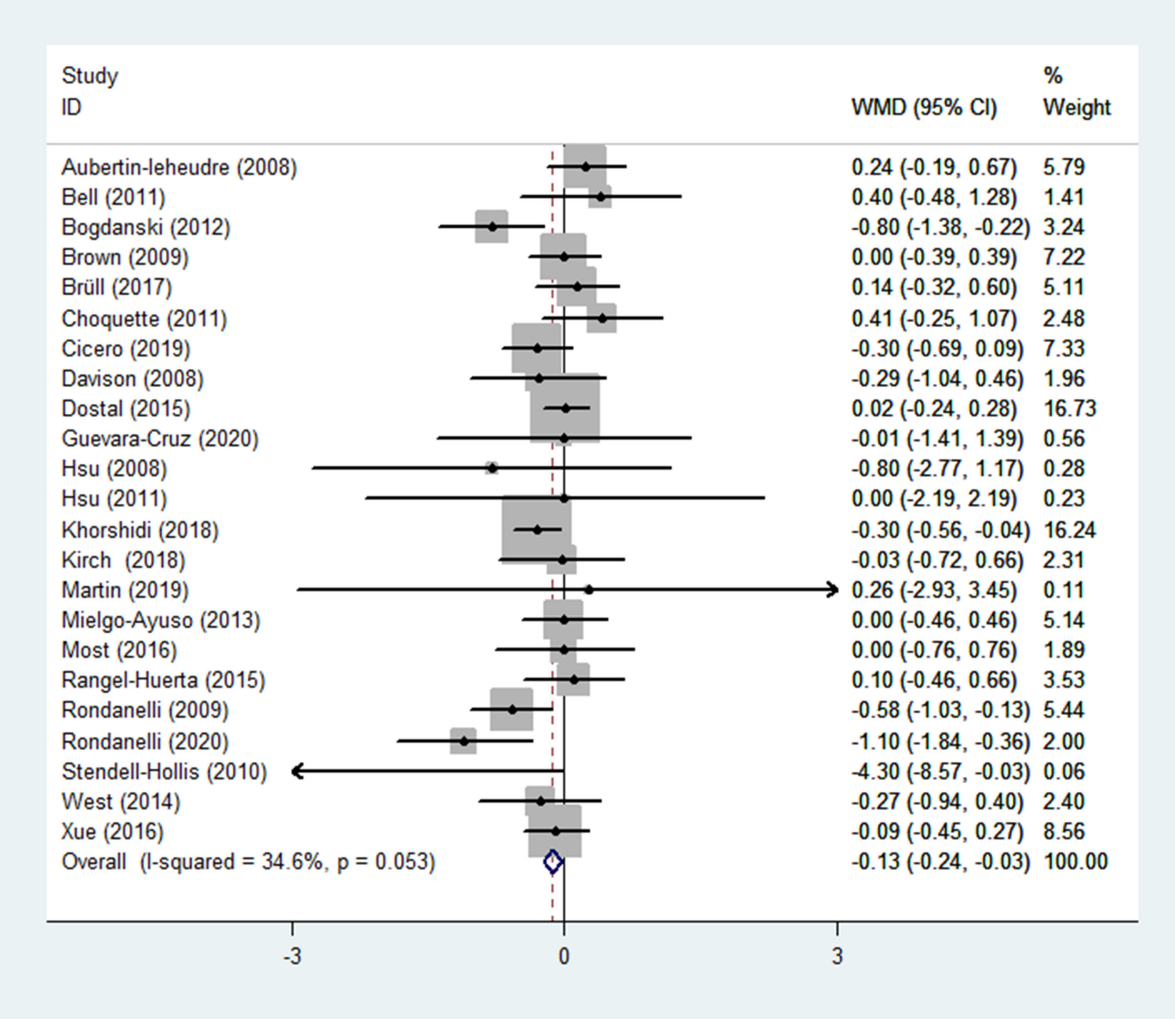
Effects of flavonoids-containing supplements on HOMA-IR in overweight and obese subjects.

The HOMA-IR was reported as an outcome in 23 RCTs (14–33, 35–37) (n = 1670), with little heterogeneity (*p* = 0.053; I^2^ = 34.6%). The findings of a fixed-effects model demonstrated that HOMA-IR was significantly lower in the experimental group (receiving flavonoids-containing supplements) compared to that in the control group (WMD = -0.13, 95% CI: -0.24 to -0.03, *p* = 0.013) (**Figure 3**).

Subgroup analyses were performed based on the interventions (whether the application of singly-used flavonoids or flavonoid-containing mixtures). In 14 RCTs (14, 16–19, 21–27, 29, 35) (n = 1106), flavonoids were singly used for interventions, results of subgroup analysis obtained with a fixed-effects model revealed that the HOMA-IR in the experimental group and the control group did not differ (WMD = -0.08, 95% CI: -0.20 to 0.05, *p* = 0.240) with low heterogeneity (*p* = 0.116, I^2^ = 32.4%) (**Table 2**). In 9 RCTs (15, 20, 28, 30–33, 36, 37) (n = 564), flavonoid-containing mixtures were used for interventions, subgroup analyses by a fixed-effects model showed that in the experimental group, HOMA-IR was significantly decreased compared to that in the control group (WMD = -0.25, 95% CI: -0.43 to -0.06, *p* = 0.008) with low heterogeneity (*p* = 0.143, I^2^ = 34.3%) (**Table 2**).

**Table 2:** Subgroup analysis of the HOMA-IR outcome.

Subgroup analyses		No. of trial	WMD	95% CI	*p*	*p; I^2^*	Effect model
Singly-used flavonoids or flavonoid-containing mixtures	Singly-used flavonoids	14	-0.08	-0.20 to 0.05	0.240	0.116; 32.4%	FE
	Flavonoid-containing mixtures	9	-0.25	-0.43 to -0.06	0.008	0.143; 34.3%	FE
Principal subclasses of flavonoids	Isoflavones	4	0.29	-0.04 to 0.62	0.082	0.937; 0.0%	FE
	Flavan-3-ols	12	-0.15	-0.31 to -0.00	0.049	0.155; 29.7%	FE
	Flavonols	2	-0.12	-0.55 to 0.30	0.568	0.103; 62.3%	RE
	Flavanones	2	-0.03	-0.33 to 0.27	0.821	0.572; 0.0%	FE
	Multiple subclasses	3	-0.46	-0.80 to -0.12	0.008	0.154; 46.6%	FE
Duration	< 12 weeks	7	-0.26	-0.48 to -0.03	0.025	0.077; 47.4%	FE
	>= 12 weeks	16	-0.10	-0.21 to 0.02	0.105	0.146; 27.6%	FE
Route of administration	Taking capsules	18	-0.12	-0.23 to -0.00	0.045	0.043; 39.7%	FE
	Taking pills	1	-0.30	-0.69 to 0.09	0.127	/	FE
	Drinking beverage	4	-0.14	-0.51 to 0.23	0.456	0.199; 35.6%	FE

WMD, weighted mean difference; CI, confidence interval; FE, fixed-effects model; RE, random-effects model.

Furthermore, subgroup analyses were performed according to principal subtypes of flavonoids. The results showed that the HOMA-IR of the experimental group significantly decreased compared to that of the control group in the subgroup receiving flavan-3-ols and the subgroup receiving multiple subclasses of flavonoids (WMD = -0.15, 95% CI: -0.31 to -0.00, *p* = 0.049; WMD = -0.46, 95% CI: -0.80 to -0.12, *p* = 0.008; respectively); however, in other subgroups, the HOMA-IR in the experimental group and the control group showed no difference (**Table 2**). The subgroup analyses were also performed based on duration, and results showed that the HOMA-IR significantly decreased in the short-duration subgroup (< 12 weeks) rather than in the long-duration subgroup (>= 12 weeks) (WMD = -0.26, 95% CI: -0.48 to -0.03, *p* = 0.025; WMD = -0.10, 95% CI: -0.21 to 0.02, *p* = 0.105; respectively) (**Table 2**).

Subgroup analyses of the routes of administration showed that, for the subgroup of taking capsules, the HOMA-IR in the experimental group significantly decreased compared to that of the control group (WMD = -0.12, 95% CI: -0.23 to -0.00, *p* = 0.045); however, for the subgroups of taking pills and drinking beverage, HOMA-IR showed no difference in the experimental group and the control group (WMD = -0.30, 95% CI: -0.69 to 0.09, *p* = 0.127; WMD = -0.14, 95% CI: -0.51 to 0.23, *p* = 0.456; respectively) (**Table 2**).

(2) QUICKI

**Figure 4:**
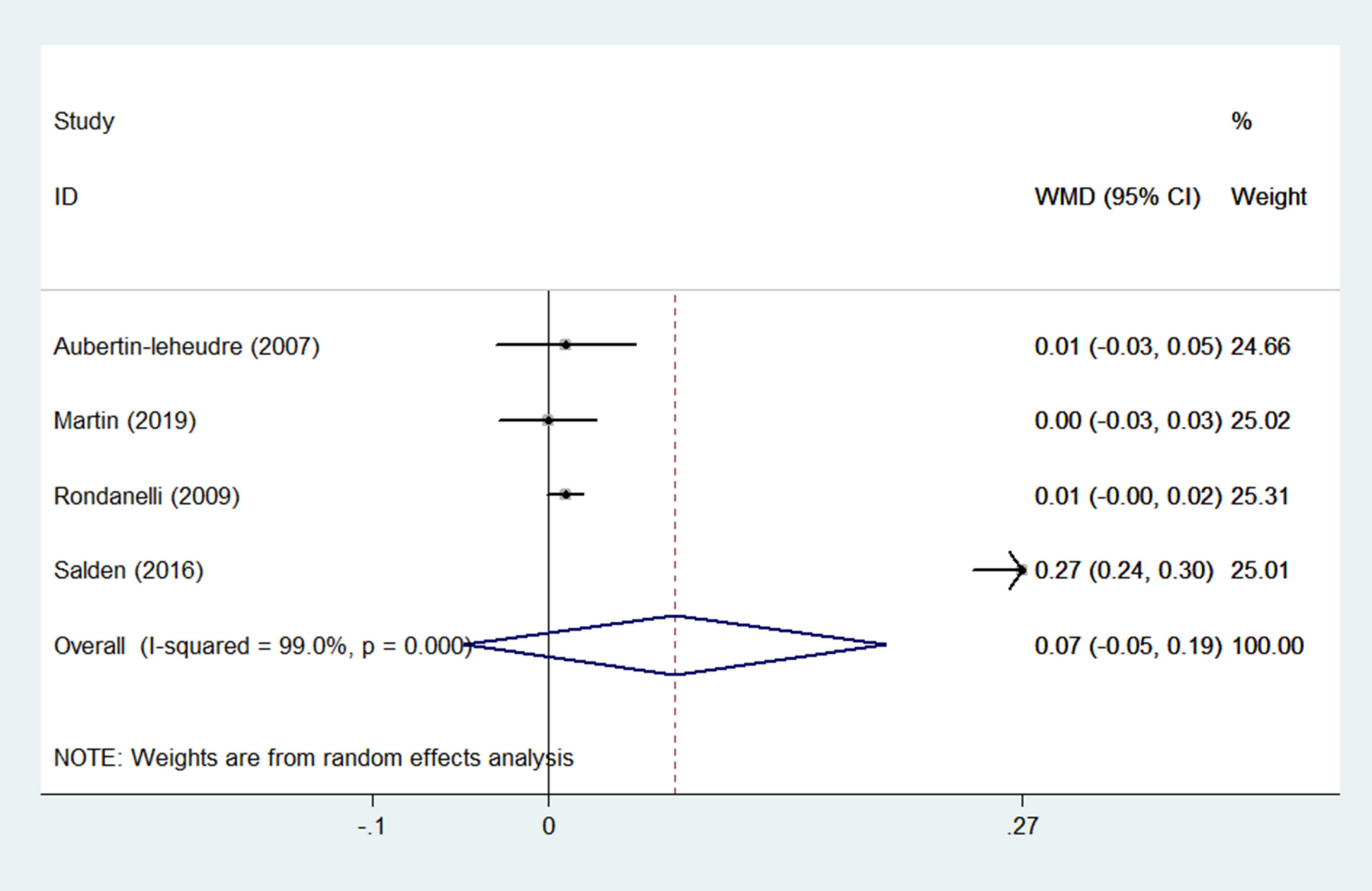
Effects of flavonoids-containing supplements on QUICKI in overweight and obese subjects.

QUICKI was reported as an outcome in 4 RCTs (13, 28, 32, 34) (n = 227), with no difference found between the experimental and control groups, according to pooled data from a random-effects model (WMD = 0.07, 95% CI: -0.05 to 0.19, *p* = 0.236) with high heterogeneity (*p* < 0.001; I^2^ = 99.0%) (**Figure 4**). The heterogeneity statistically decreased (*p* = 0.801, I^2^ = 0.0%) after excluding Salden et al. (34), and pooled results showed that the QUICKI in the experimental group had an increasing trend compared to the control group, and the data were not statistically different (WMD = 0.01, 95% CI: -0.00 to 0.02, *p* = 0.065) (**Table 3**).

**Table 3:** Results of sensitivity analysis.

Outcomes	Excluded RCTs	Remaining RCTs	WMD	95% CI	*p*	*p; I^2^*	Effect model
QUICKI	Salden et al., 2016	3	0.01	-0.00 to 0.02	0.065	0.801; 0.0%	FE
LDL-C	Cicero et al., 2019; Davison et al., 2008	20	-0.02	-0.04 to 0.00	0.100	0.676; 0.0%	FE
DBP	Bogdanski et al., 2012	14	-0.57	-1.56 to 0.43	0.264	0.147; 28.9%	FE

RCTs, randomized controlled trials; WMD, weighted mean difference; CI, confidence interval; QUICKI, quantitative insulin sensitivity check index; LDL-C, low-density lipoprotein cholesterol; DBP, diastolic blood pressure; FE, fixed-effects model.

#### 3.4.2 Secondary outcomes

(1) FBG

**Figure 5:**
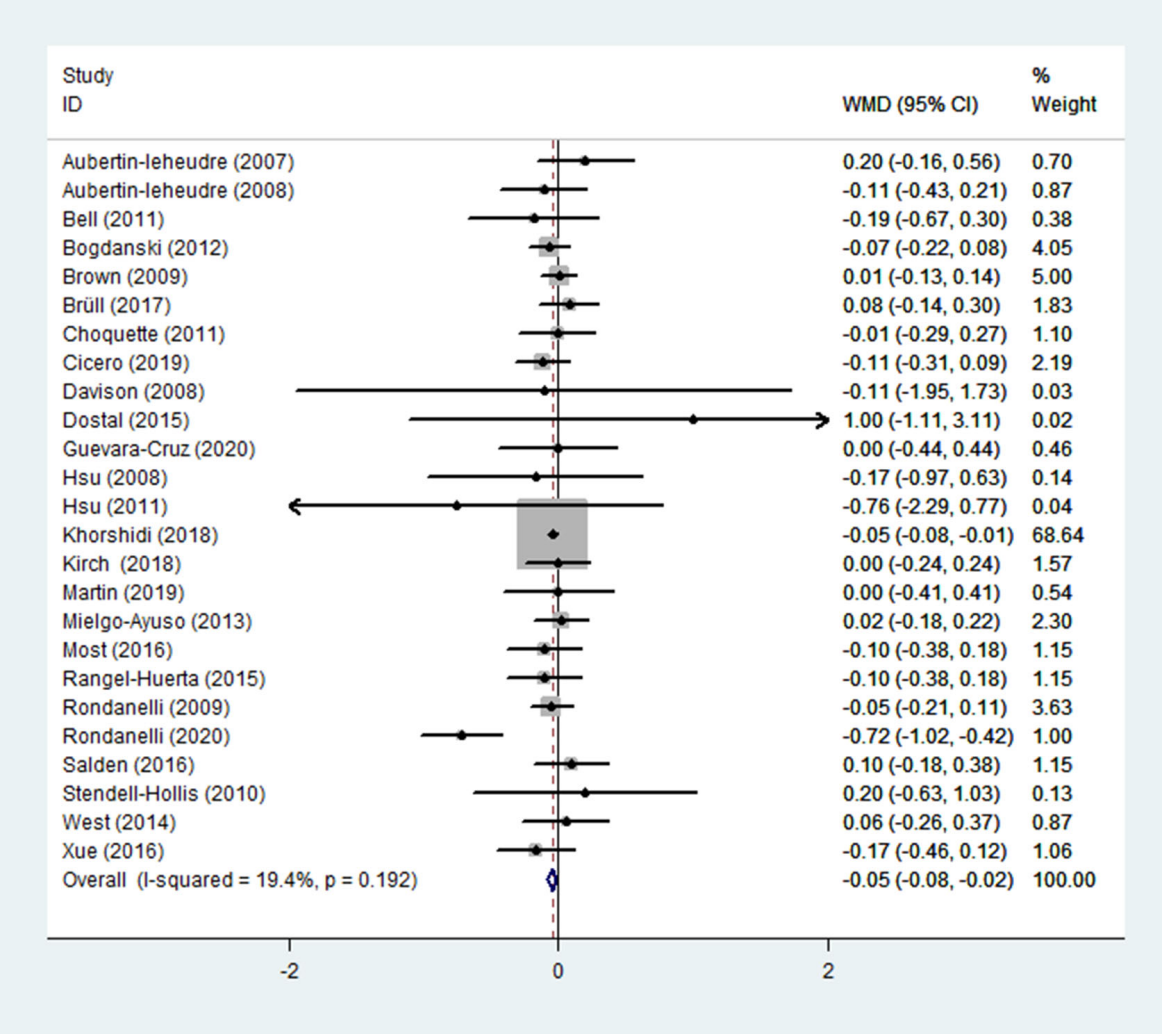
Effects of flavonoids-containing supplements on fasting blood glucose.

FBG was reported as an outcome in 25 trials (13–37) (n = 1755), in which low heterogeneity was found (*p* = 0.192; I^2^ = 19.4%). FBG levels in the experimental group (receiving flavonoid-containing supplements) were significantly lower than in the control group, according to pooled data from a fixed-effects model (WMD = -0.05, 95% CI: -0.08 to -0.02, *p* = 0.002) (**Figure 5**).

(2) FBI

**Figure 6:**
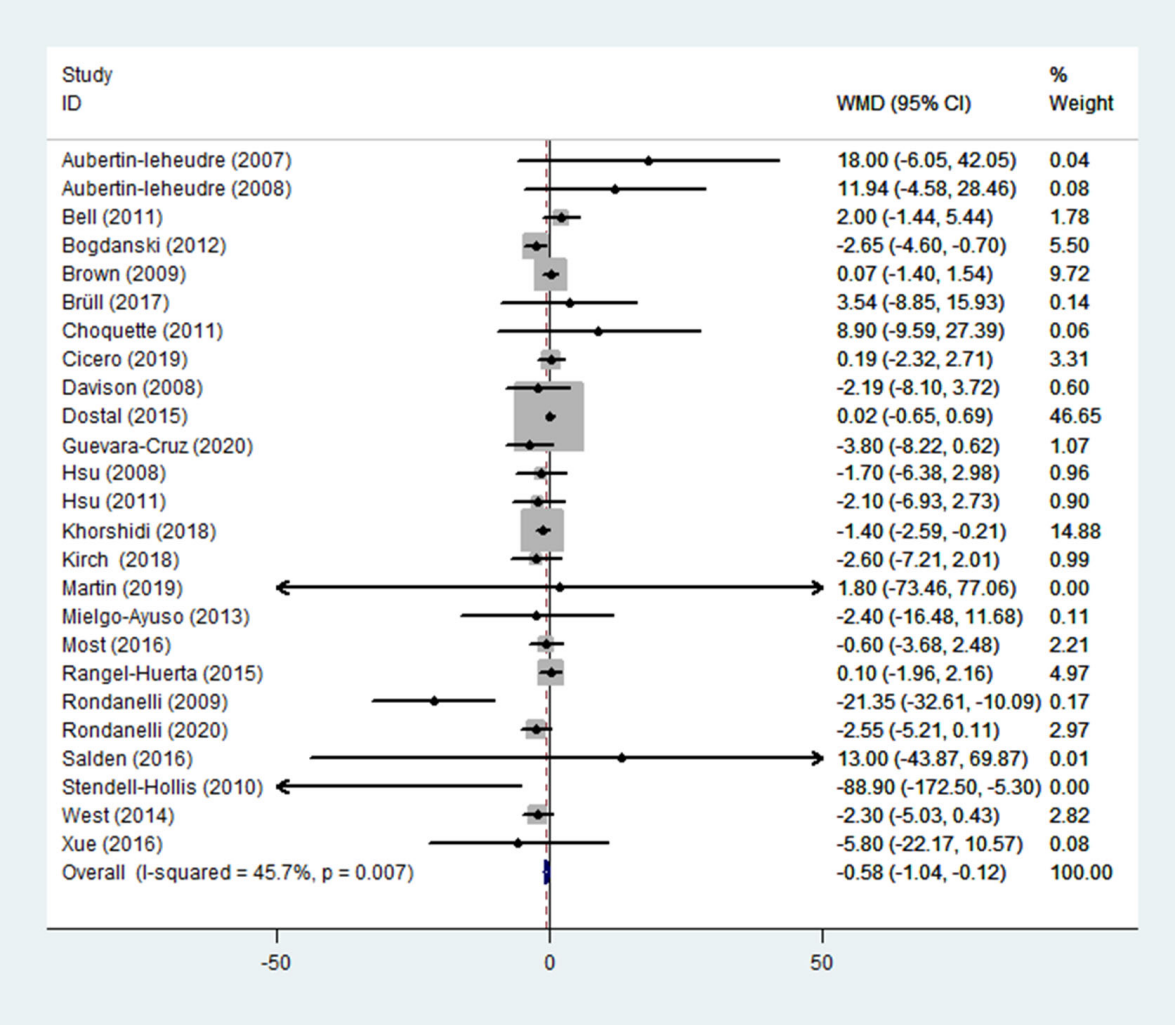
Effects of flavonoids-containing supplements on fasting blood insulin.

FBI was reported in 25 RCTs (13–37) (n = 1747), in which moderate heterogeneity was observed (*p* = 0.007; I^2^ = 45.7%). Results from a fixed-effects model demonstrated that in the experimental group (receiving flavonoids-containing supplements), the FBI significantly reduced compared to that in the control group (WMD = -0.58, 95% CI: -1.04 to -0.12, *p* = 0.014) (**Figure 6**).

(3) Blood lipids

Blood lipids including plasma total cholesterol (TC), triglycerides (TG), high-density lipoprotein cholesterol (HDL-C), and low-density lipoprotein cholesterol (LDL-C) were reported in 22 RCTs (13–17, 19–21, 23–25, 27–37) (n = 1305). Results of meta-analysis obtained with a fixed-effects model demonstrated that in the group receiving flavonoids-containing supplements, TC and TG significantly decreased compared to that in the controls (WMD = -0.04, 95% CI: -0.06 to -0.03, *p* < 0.001; WMD = -0.04, 95% CI: -0.05 to -0.03, *p* < 0.001; respectively) with moderate heterogeneity (*p* = 0.039, I^2^ = 37.8%; *p* = 0.019, I^2^ = 42.5%; respectively) (**Table 4**). However, HDL-C did not differ between the two groups (WMD = 0.01, 95% CI: -0.00 to 0.02, *p* = 0.143) with moderate heterogeneity (*p* = 0.025, I^2^ = 40.9%) (**Table 4**). Furthermore, LDL-C did not differ between the two groups (WMD = -0.26, 95% CI: -0.54 to 0.03, *p* = 0.078) with obvious heterogeneity (*p* < 0.001, I^2^ = 95.6%) (**Table 4**). Results of sensitivity analyses revealed that after excluding Cicero et al. (20) and Davison et al. (21), heterogeneity was significantly reduced (*p* = 0.676, I^2^ = 0.0%), and results still showed that the LDL-C in the experimental group and the control group did not differ (WMD = -0.02, 95% CI: -0.04 to 0.00, *p* = 0.100) (**Table 3**).

**Table 4:** Meta-analysis for the outcomes of blood lipids, blood pressure, and adverse effects.

Outcomes	No. of trial	WMD/RR	95% CI	*p*	*p; I^2^*	Effect model
TC	22	-0.04	-0.06 to -0.03	< 0.001	0.039; 37.8%	FE
TG	22	-0.04	-0.05 to -0.03	< 0.001	0.019; 42.5%	FE
HDL-C	22	0.01	-0.00 to 0.02	0.143	0.025; 40.9%	FE
LDL-C	22	-0.26	-0.54 to 0.03	0.078	< 0.001; 95.6%	RE
SBP	15	-2.01	-3.17 to -0.86	0.001	0.410; 3.8%	FE
DBP	15	-0.82	-2.23 to 0.60	0.257	0.010; 52.1%	RE
Adverse effects	14	0.97	0.62 to 1.52	0.905	0.533; 0.0%	FE

WMD, weighted mean difference; RR, relative risk; CI, confidence interval; TC, total cholesterol; TG, triglycerides; HDL-C, high-density lipoprotein cholesterol; LDL-C, low-density lipoprotein cholesterol; SBP, systolic blood pressure; DBP, diastolic blood pressure; FE, fixed-effects model; RE, random-effects model.

(4) Blood pressure

Systolic blood pressure (SBP) and diastolic blood pressure (DBP) were presented in 15 RCTs (14–17, 20, 21, 23–25, 27, 28, 31, 33, 34, 37) (n = 909). Pooled results of a fixed-effects model revealed that in the group receiving flavonoids-containing supplements, SBP significantly decreased compared to that in the control group (WMD = -2.01, 95% CI: -3.17 to -0.86, *p* = 0.001) with low heterogeneity (*p* = 0.410, I^2^ = 3.8%) (**Table 4**). However, DBP in the experimental group and the control group did not differ (WMD = -0.82, 95% CI: -2.23 to 0.60, *p* = 0.257) with obvious heterogeneity (*p* = 0.010, I^2^ = 52.1%) (**Table 4**). Results of sensitivity analyses showed that after excluding Bogdanski et al. (16), heterogeneity significantly dropped (*p* = 0.147, I^2^ = 28.9%), and pooled results still showed that DBP in the experimental group and the control group did not differ (WMD = -0.57, 95% CI: -1.56 to 0.43, *p* = 0.264) (**Table 3**).

(5) Weight

**Figure 7:**
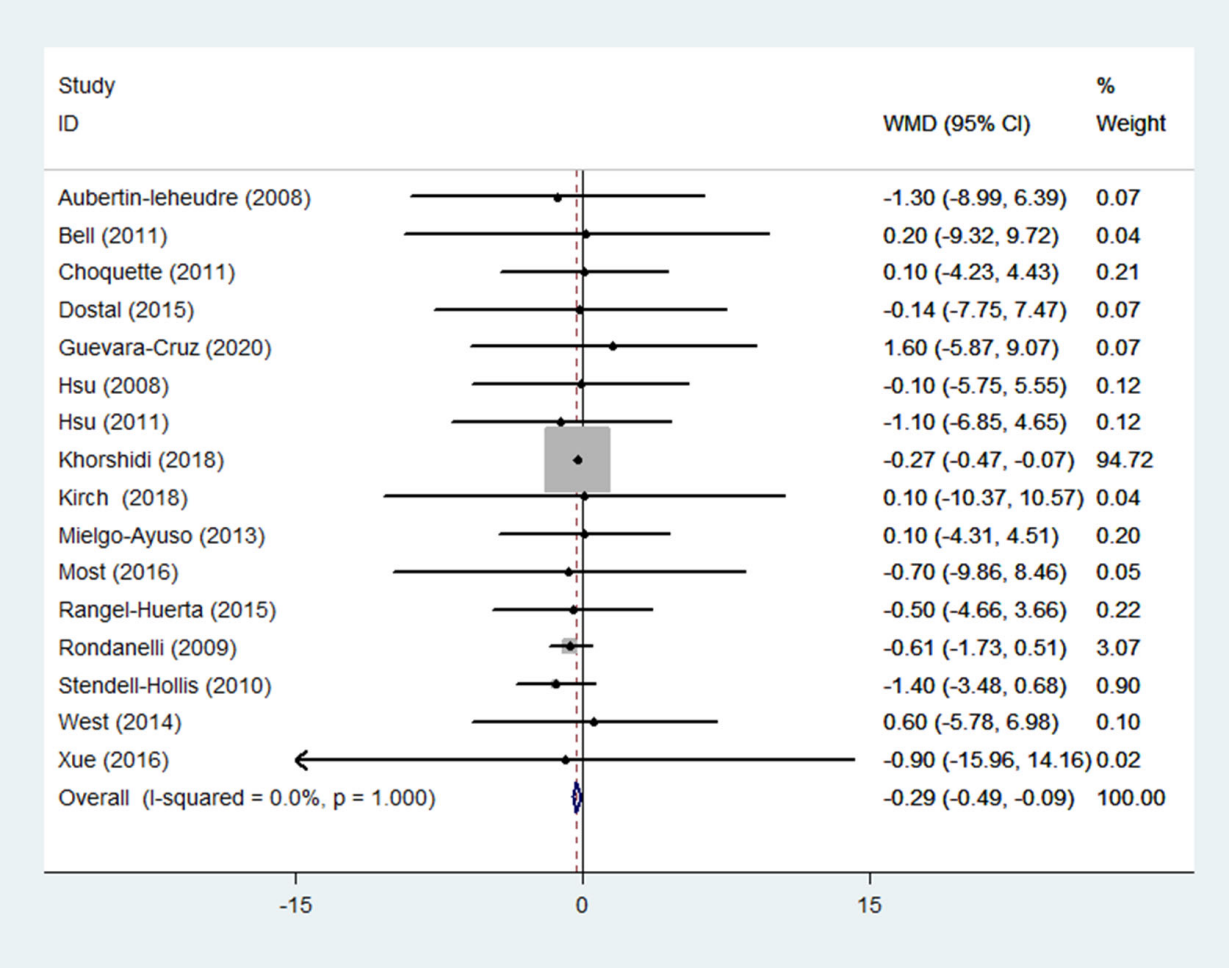
Effects of flavonoids-containing supplements on weight.

Weight was reported as an outcome in 16 RCTs (14, 15, 19, 22–27, 29–32, 35–37) (n = 1166). Pooled results from a fixed-effects model demonstrated that in the group receiving flavonoids-containing supplements, weight significantly decreased compared to that in the control group (WMD = -0.29, 95% CI: -0.49 to -0.09, *p* = 0.004) with low heterogeneity (*p* = 1.000, I^2^ = 0.0%) (**Figure 7**).

(6) BMI

**Figure 8:**
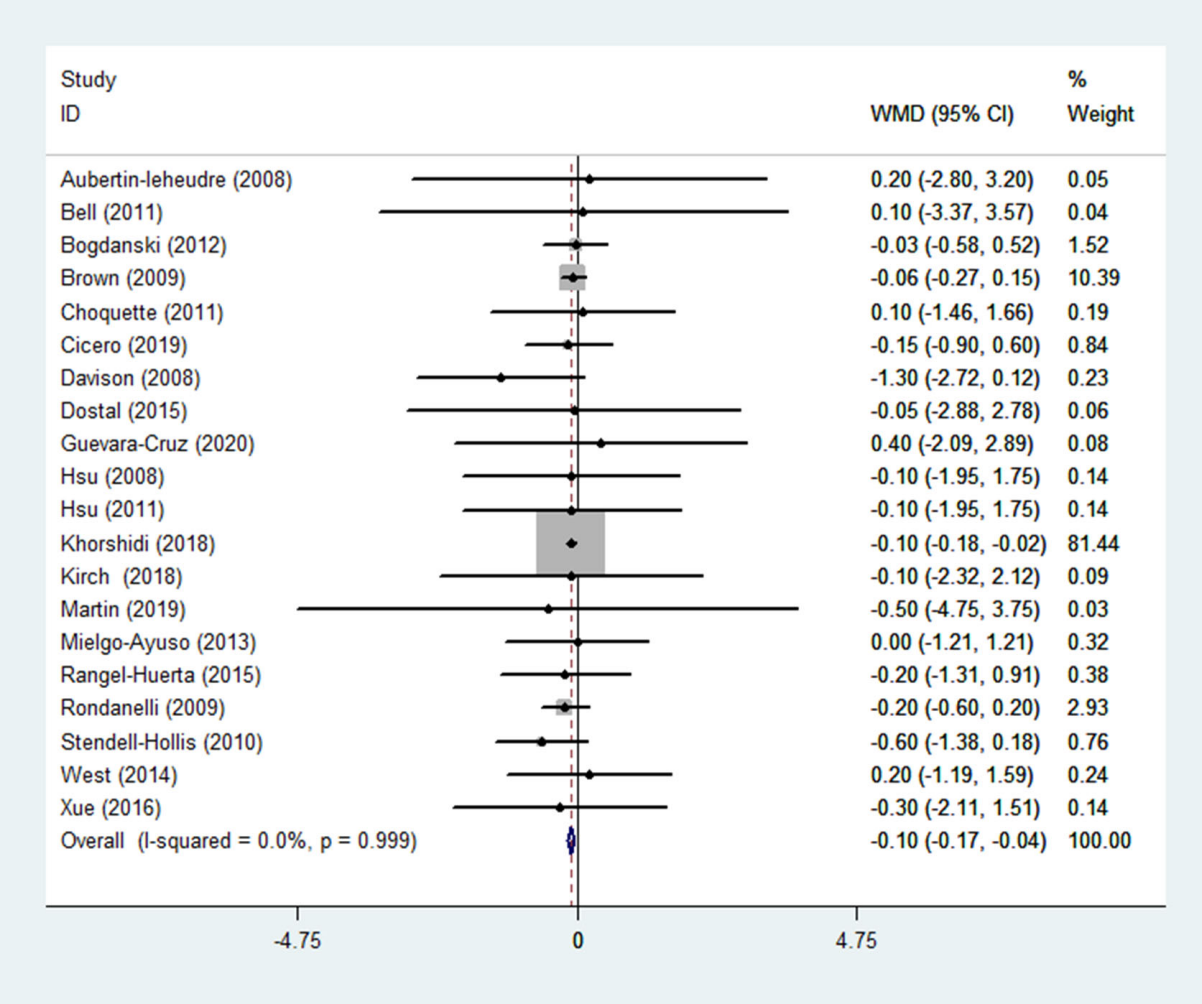
Effects of flavonoids-containing supplements on body mass index.

BMI was reported in 20 RCTs (14–17, 19–29, 31, 32, 35–37) (n = 1411). Pooled results obtained from a fixed-effects model indicated that in the group receiving flavonoids-containing supplements, BMI significantly decreased compared to that in the control group (WMD = -0.10 95% CI: -0.17 to -0.04, *p* = 0.003) with low heterogeneity (*p* = 0.999, I^2^ = 0.0%) (**Figure 8**).

(7) WC

**Figure 9:**
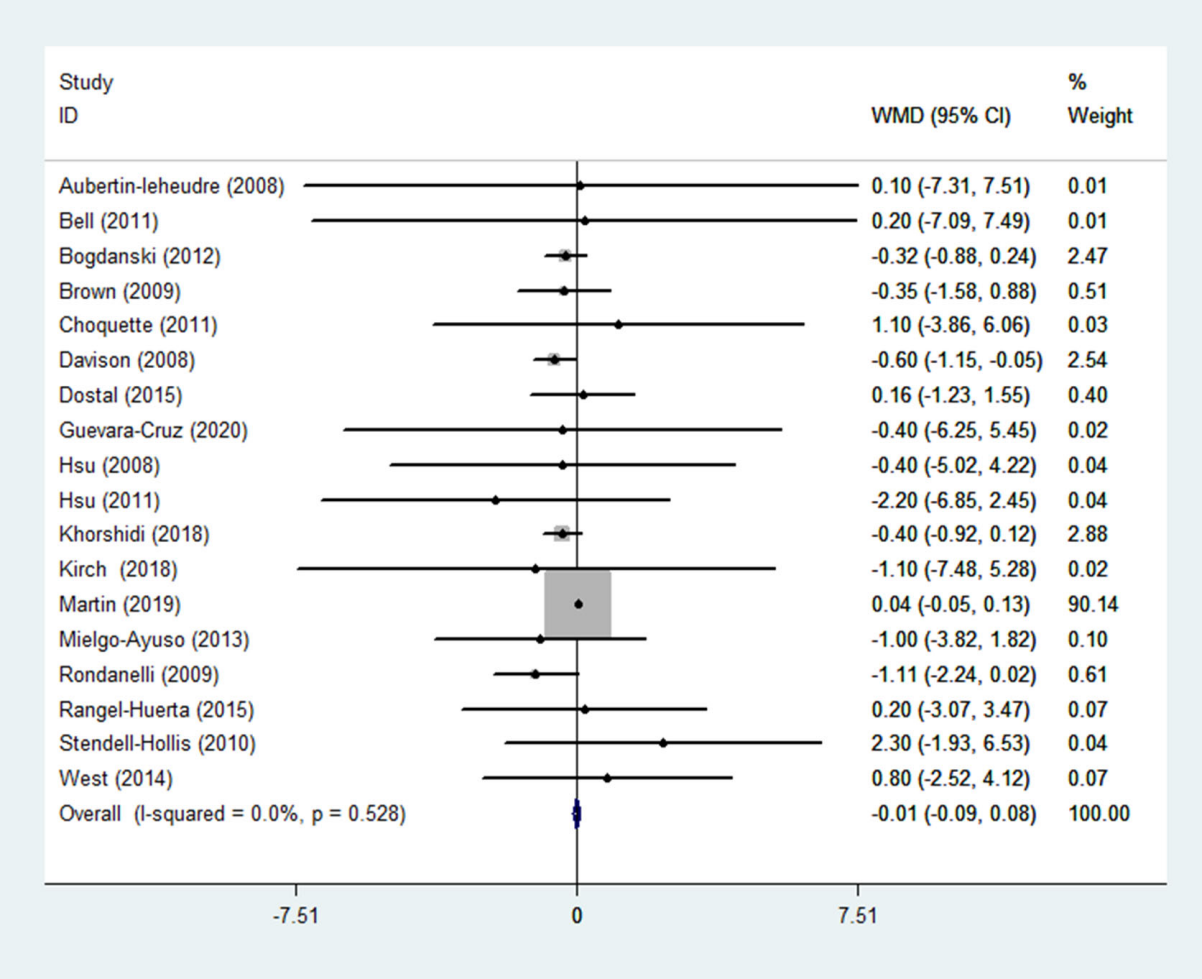
Effects of flavonoids-containing supplements on waist circumference.

WC was reported in 18 RCTs (14–17, 19, 21–29, 31, 32, 35, 36) (n = 1299). Pooled results obtained with a fixed-effects model showed that WC in the group receiving flavonoids-containing supplements and the control group did not differ (WMD = -0.01, 95% CI: -0.09 to 0.08, *p* = 0.883) with low heterogeneity (*p* = 0.528, I^2^ = 0.0%) (**Figure 9**).

(8) WHR

**Figure 10:**
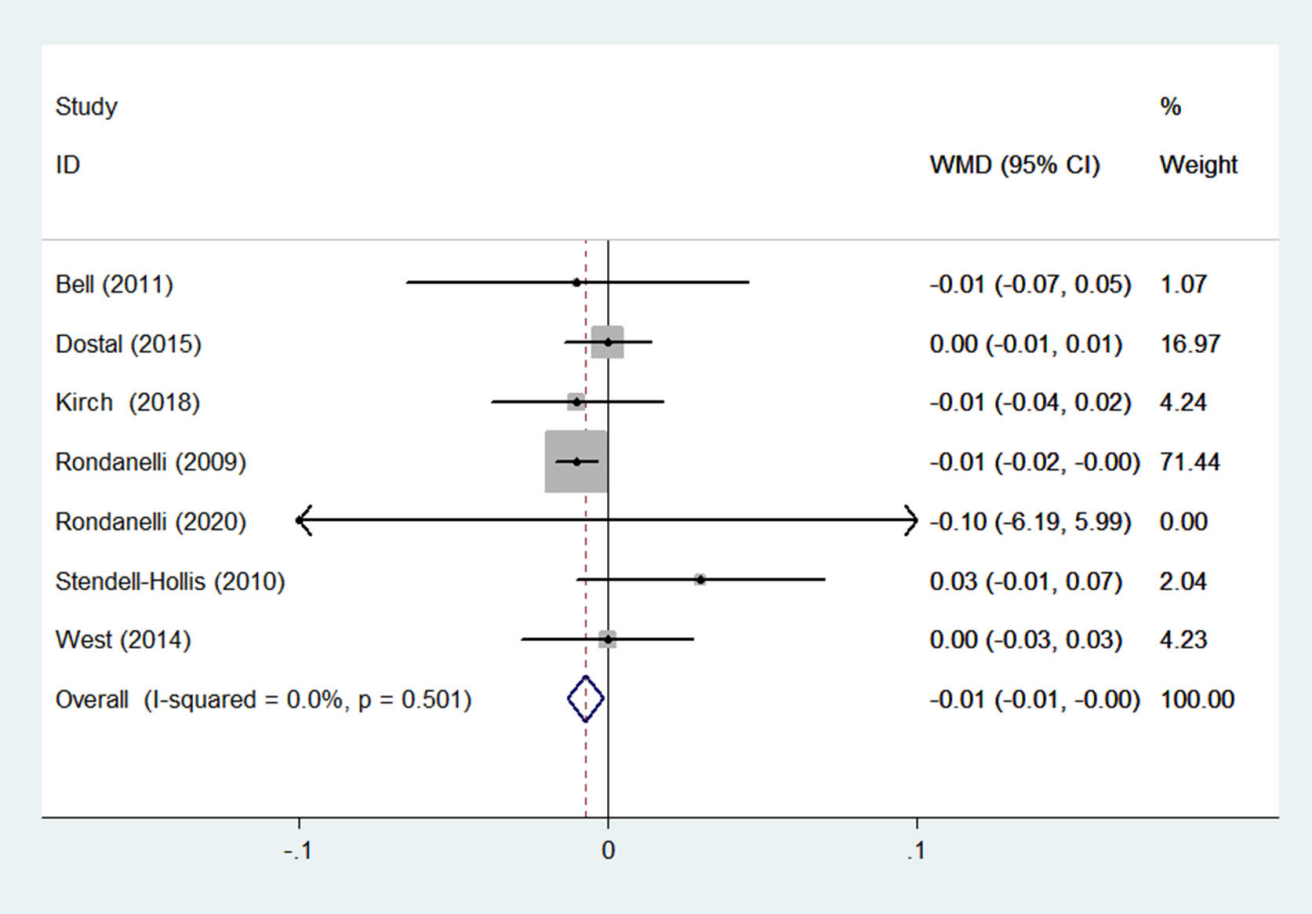
Effects of flavonoids-containing supplements on waist-to-hip ratio.

WHR was reported in 7 RCTs (15, 22, 27, 32, 33, 35, 36) (n = 624). Results obtained from a fixed-effects model demonstrated that in the group receiving flavonoids-containing supplements, WHR was significantly lower compared to that in the control group (WMD = -0.01, 95% CI: -0.01 to -0.00, *p* = 0.015) with small heterogeneity (*p* = 0.501, I^2^ = 0.0%) (**Figure 10**).

(9) Adverse effects

In 14 RCTs (13, 15, 18–20, 22, 24–26, 29, 30, 32, 33, 37) (n = 1130), adverse effects were reported as an outcome. Among 10 of them, no adverse effects were found in the group receiving flavonoid-containing supplements or the control group. Pooled results demonstrated that in the group receiving flavonoid-containing supplements, adverse reactions did not increase compared to that in the control group (RR = 0.97, 95% CI: 0.62 to 1.52, *p* = 0.905) with no heterogeneity (*p* = 0.533; I^2^ = 0.0%) (**Table 4**).

### 3.5 Publication bias

**Figure 11:**
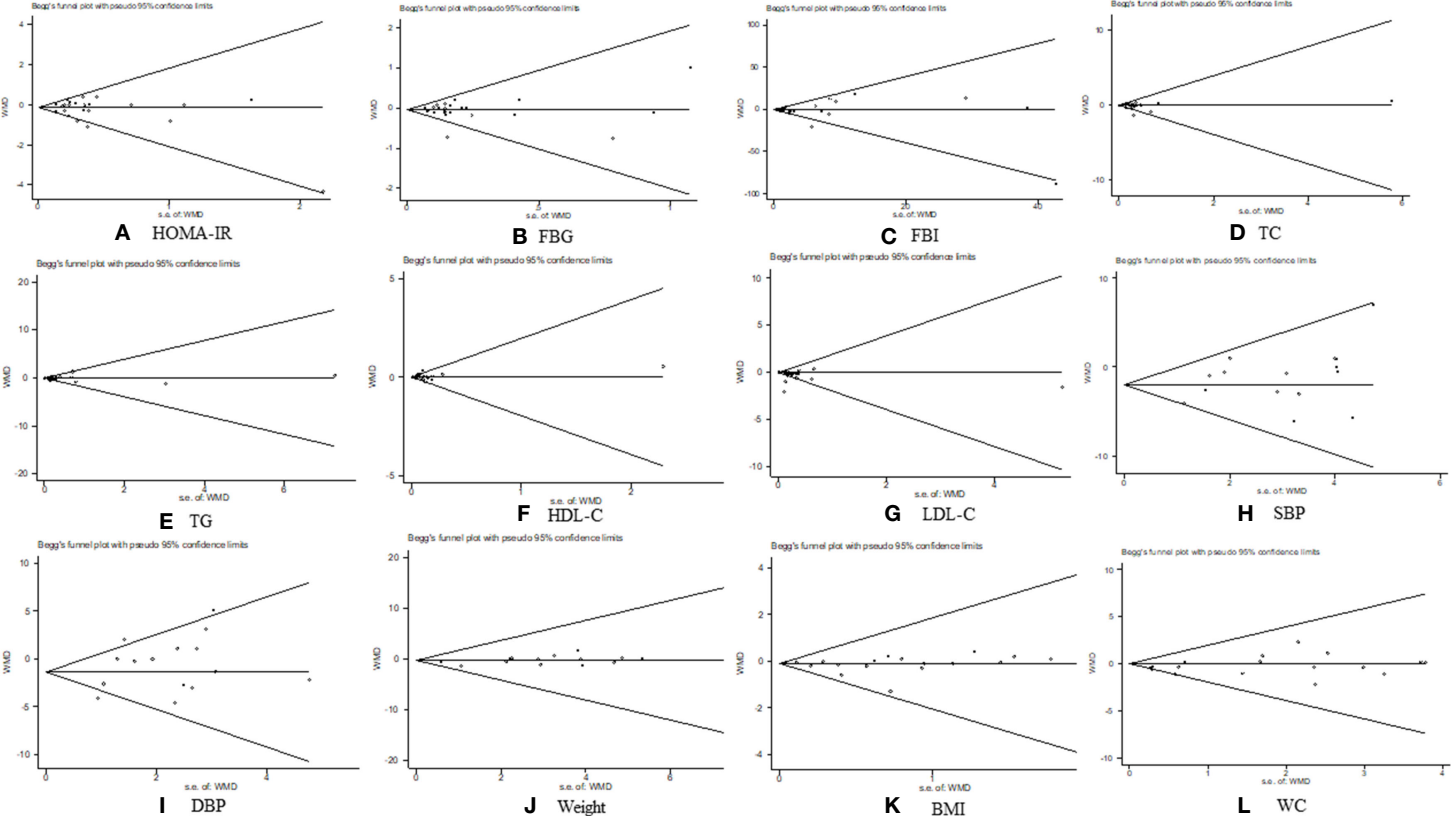
Publication bias analysis. **(A)** homeostasis model assessment of insulin resistance, **(B)** fasting blood glucose **(C)** fasting blood insulin, **(D)** total cholesterol, **(E)** triglycerides, **(F)** high-density lipoprotein cholesterol, **(G)** low-density lipoprotein cholesterol, **(H)** systolic blood pressure, **(I)** diastolic blood pressure, **(J)** weight, **(K)** body mass index, and **(L)** waist circumference.

The outcomes of HOMA-IR, FBG, FBI, TC, TG, HDL-C, LDL-C, SBP, DBP, weight, BMI, and WC were evaluated based on a publication bias analysis. Most scatter points fell within the confidence limit, and the funnel plots were symmetrical. The *p*-value of Begg’s tests were 0.342, 0.591, 0.657, 0.535, 0.236, 0.535, 0.693, 0.488, 0.488, 0.893, 0.922, and 0.198, respectively; that of Egger’s tests were 0.483, 0.802, 0.234, 0.261, 0.677, 0.833, 0.175, 0.133, 0.120, 0.552, 0.422, and 0.106, respectively (**Figure 11**). The results of the publication bias analysis revealed that none of the included trials in the aforementioned outcome indicators had any potential of publication bias.

## 4 Discussion

The current study firstly analyzed extant data and found that flavonoids-containing supplements might be effective and safe in treating IR and related metabolic risk factors (such as FBG, FBI, TC, TG, SBP, weight, BMI, and WHR) in overweight and obese subjects, based on 25 RCTs. This study provided preliminary evidence for the use of flavonoid-containing supplements as a treatment option for overweight and obesity.

For HOMA-IR, results demonstrated that HOMA-IR in the group receiving flavonoid-containing supplements significantly decreased versus the control group. Subgroup analyses showed that the effect of flavonoid-containing supplements on reducing HOMA-IR might be related to the interventions (singly-used flavonoids or flavonoid-containing mixtures). In the present study, flavonoids were singly used for interventions (isoflavone, EGCG, quercetin, flavanol, genistein, green tea extract, (–)-epicatechin, hesperidin 2S, and catechin) in 14 RCTs. Furthermore, flavonoid-containing mixtures (with flavonoids as the dominant compositions) were used for interventions in 9 RCTs. The RCT conducted by Bell et al. (15) used Glavonoid™ as an intervention, with the main ingredients composed of 30% licorice glabra polyphenol and 3% glabridin, both of which belong to glavonoid. Cicero et al. (20) used bergamot extract (120 mg flavonoids per pill) as intervention. Martin et al. (28) used tart cherry juice with anthocyanins and phenolics as main ingredients. Most et al. (30) used the co-formulation of EGCG and resveratrol, with EGCG (282 mg daily) as the main ingredient. Rangel-Huerta et al. (31) used polyphenol, with hesperidin and narirutin as the main ingredients. Rondanelli et al. (32) used the co-formulation of N-oleyl-phosphatidylethanolamine and EGCG. The amelioration of IR could be attributed to EGCG (100 mg daily), and the effect of N-oleyl-phosphatidylethanolamine mainly included reduction of food intake and amelioration *in vivo* plasma availability of EGCG (38). Rondanelli et al. (33) used *Cynara*, of which flavonoids (>= 1.5%) were the main component, and it was only lower than that of caffeoylquinic acids. West et al. (36) used cocoa/chocolate containing flavanols (814 mg daily). Xue et al. (37) used trans-resveratrol-hesperetin co-formulation, and the main ingredient was hesperetin (120 mg daily). In the singly-used flavonoids subgroup, HOMA-IR in the experimental group and the control group did not differ. However, in the subgroup receiving flavonoid-containing mixtures, HOMA-IR significantly decreased in the experimental group compared to that in the control group. Pooled results showed that QUICKI between the experimental group and the control group did not differ. However, the results may be influenced by the obvious heterogeneity (I^2^ = 99.0%). Salden et al. (34) used hesperidin 2S as an intervention for the experimental group, the sensitivity analyses showed that after excluding Salden et al. (34), the heterogeneity statistically decreased (I^2^ = 0.0%), pooled results indicated that QUICKI in the experimental group had an increasing trend compared to that in the control group. Similar to our findings, a series of comprehensive reviews also reported the effects of flavonoids on alleviating IR (39–41). Furthermore, according to a meta-analysis conducted of type 2 diabetes mellitus subjects conducted by Liu et al. (42), flavonoids brought significant benefits to glucose metabolism and insulin sensitivity, especially significantly lowing FBG, HOMA-IR, and HbA1c. Another meta-analysis reported the beneficial effects of flavan-3-ol intake on cardiometabolic outcomes including HOMA-IR (43).

Flavonoids have a variety of physiologic properties, including anti-inflammatory and antioxidative activities. They may help to reduce IR (9) by blocking the formation and expression of proinflammatory mediators and/or enzymes, such as suppressing inflammatory cytokines *via* the TLR4/NF-B signaling pathway, stimulating AMPK, activating autophagy, and protecting against the atrophy of obesity-related skeletal muscle by repressing inflammatory cytokines and macrophage infiltration (44). Additionally, flavonoids may regulate whole-body glucose homeostasis by interacting with a variety of molecular targets in the small intestine, pancreas, skeletal muscle, adipose tissue, and liver. Flavonoids also exhibit pleiotropic properties such as decreasing intestinal glucose absorption, improving insulin secretory and insulin-sensitizing actions, and increasing glucose consumption in peripheral tissues, all of which contribute to improving IR (9). However, in the present study, HOMA-IR did not show a difference in the singly-used flavonoids group compared to that in the control group and the QUICKI in the experimental group had an increasing trend compared to that in the control group, probably because of different subtypes of flavonoids and the limited RCTs included. In addition, apart from flavonoids, the above flavonoid-containing mixtures may contain other ingredients (such as phenolics and caffeoylquinic acids) that may be beneficial to reduce IR.

Furthermore, subgroup analyses based on principal subclasses of flavonoids showed that HOMA-IR significantly decreased in the experimental group versus the control group when using flavan-3-ols and multiple subclasses of flavonoids. Thus, we speculated that different subclasses of flavonoids might have different potencies in reducing HOMA-IR. Flavan-3ols are the most frequently used flavonoids in the diet, and they are found in drinks, fruits, vegetables, grains, herbal medicines, nutritional supplements, and dairy products. Catechin, epicatechin, catechin gallate, gallocatechin, epigallocatechin, epicatechin gallate, gallocatechin gallate, and EGCG are the major components of flavan-3-ols. Flavan-3-ols, particularly EGCG, have been linked to hypoglycemic, anti-inflammatory, antioxidant, and thermogenic activities (45). Various *in vivo* or *in vitro* experimental studies on catechins and their chemical derivatives have reported that they can improve IR. There are four hypothesized mechanisms: 1) suppressing the inflammatory pathway mediated by NF-kappa B (46, 47); 2) reducing free radicals *via* inhibiting lipid peroxidation, stimulating antioxidant enzymes (48), suppressing redox-sensitive transcription factors, and decreasing pro-oxidant enzyme mechanisms (49); 3) stimulating pancreatic beta cells to improve postprandial insulin, thereby improving pancreas function (50); 4) reducing adipocyte proliferation and differentiation while improving glucose receipt by the cells *via* protein kinase activation, a mechanism similar to that utilized by hypoglycemic medicines such as metformin (24, 51). Our findings suggest that various flavonoid subclasses may have varying potencies in enhancing IR; nevertheless, results remain uncertain because by far no direct comparative studies have been conducted.

As for secondary outcomes, pooled results demonstrated that in the group receiving flavonoid-containing supplements, other metabolic markers including FBG, FBI, TC, TG, SBP, weight, BMI, and WHR significantly decreased compared to those in the controls. Other studies also showed that flavonoids can prevent and/or ameliorate obesity and obesity-associated diseases (52, 53). IR is thought to be the common core pathogenic foundation of metabolic diseases such as metabolic syndrome, hypertension, and diabetes mellitus, all of which endanger human health (54, 55). As mentioned above, flavonoids have the effect of improving insulin resistance, which is beneficial to preventing and/or ameliorating obesity and obesity-associated diseases (such as diabetes mellitus, hypertension, dyslipidemia, and metabolic syndrome). Some other mechanisms by which flavonoids improve obesity and obesity-associated diseases have also been found (52). Studies indicated that many flavonoids can reduce oxidative stress, enhance glucose tolerance, alter lipid metabolism and adipocyte differentiation, inhibit inflammation and apoptosis, and ameliorate endothelial dysfunction (56–60), showing that they may have an anti-diabetic effect. Furthermore, the effects of flavonoids on hypertension are well established and appear to be mechanistically connected to NO bioavailability, which is controlled by NOS activation and/or NOX inhibition. Additionally, flavonoids may reduce lipid absorption in the gastrointestinal tract, as well as help regulate the activity of various enzymes included in lipid metabolism and the expression of transcription factors included in TG and cholesterol synthesis, such as the sterol regulatory element-binding proteins SREBP-1 and SREBP-2 (61), indicating their potential anti-hyperlipidemic effect. Furthermore, aggregated data revealed that the incidence of adverse reactions did not differ between the group receiving flavonoids-containing supplements and the control group, showing that flavonoids-containing supplements were safe and well-tolerated in clinical practice under general usage settings.

Nevertheless, the present study has certain limitations which should be considered. Firstly, this study encompassed all flavonoids-containing supplements, including solely flavonoids and mixtures, but it included only a few RCTs for each type of flavonoids-containing supplement. Therefore, more RCTs are needed to identify the efficacy of each type of flavonoids-containing supplement in overweight and obese subjects. Secondly, due to the small number of included RCTs, publication bias was not assessed in the QUCIKI and WHR outcomes; more RCTs with the outcomes of QUCIKI and WHR reported are needed to further verify our conclusion. Thirdly, several of the included RCTs were of poor quality. For example, they were a single-centered study with a small number of participants. Fourthly, further study is required to quantify the optimal type and amounts of flavonoids for determining the appropriate prescription of flavonoid consumption to treat overweight and obese patients. Fifthly, given that the included RCTs were conducted in different countries and regions, more high-quality studies using uniformly sourced flavonoids-containing supplements are needed to further verify our conclusions. Furthermore, further studies on the pharmacological processes, long-term toxicity, and bioavailability of flavonoids in the treatment of overweight and obese people are needed. Given the limitations of this study, we suggest that the findings should be validated in future research.

## 5 Conclusion

This systematic review and meta-analysis evaluated the current data and demonstrated that flavonoids-containing supplements might be efficacious and safe in treating IR and related metabolic risk factors (such as FBG, FBI, TC, TG, SBP, weight, BMI, and WHR) in overweight and obese subjects. This study presented preliminary evidence to guide the use of flavonoids-containing supplements as a therapy option for overweight and obese subjects. However, there is still doubt over the findings because limited RCTs for per kind of flavonoids-containing supplement were investigated, and many of the RCTs had a small sample size and short duration. Given the limitations, we propose that the results should be established or confirmed on a larger scale with more precise instructions in future investigations.

## Data availability statement

The original contributions presented in the study are included in the article/**Supplementary Material**. Further inquiries can be directed to the corresponding author.

## Author contributions

Conceptualization: JY. Data curation: JY, YZ, JZ, X-ZW. Formal analysis: JZ. Project administration: Y-PL, G-JF, LS. Supervision: G-JF. Validation: JY, Q-YL. Writing – original draft: JY, JZ, YZ. Writing – review & editing: G-JF, LS. All the authors have read and approved the manuscript.

## Funding

This study was supported by the Chinese Government, Ministry of Science, and Technology of the People’s Republic of China through the National Science and Technology Support Program (Grant No. 2015BAI04B09) and Guangdong Provincial Hospital of Traditional Chinese Medicine (Grant No. 2021DB02).

## Acknowledgments

We thank Zhaojun Yang for preparing this manuscript.

## Conflict of interest

The authors declare that the research was conducted in the absence of any commercial or financial relationships that could be construed as a potential conflict of interest.

## Publisher’s note

All claims expressed in this article are solely those of the authors and do not necessarily represent those of their affiliated organizations, or those of the publisher, the editors and the reviewers. Any product that may be evaluated in this article, or claim that may be made by its manufacturer, is not guaranteed or endorsed by the publisher.

## Abbreviations

IR, insulin resistance; RCTs, randomized controlled trials; HOMA-IR, homeostasis model assessment of insulin resistance; WMD, weighted mean difference; CI, confidence interval; QUICKI, quantitative insulin sensitivity check index; FBG, fasting blood glucose; FBI, fasting blood insulin; TC, total cholesterol; TG, triglycerides; SBP, systolic blood pressure; BMI, body mass index; WHR, waist-to-hip ratio; RR, relative risk. **Main text section:** IR, insulin resistance; RCTs, randomized controlled trials; HOMA-IR, homeostasis model assessment of insulin resistance; QUICKI, quantitative insulin sensitivity check index; FBG, fasting blood glucose; FBI, fasting blood insulin; BMI, body mass index; WC, waist circumference; WHR, waist-to-hip ratio; WMD, weighted mean difference; CI, confidence interval; RR, relative risk; EGCG, epigallocatechin gallate; TC, total cholesterol; TG, triglycerides; HDL-C, high-density lipoprotein cholesterol; LDL-C, low-density lipoprotein cholesterol; SBP, systolic blood pressure; DBP, diastolic blood pressure.
