# Supplementary material for: Efficacy of flavonoids-containing supplements on insulin resistance and associated metabolic risk factors in overweight and obese subjects: a systematic review and meta-analysis of 25 randomized controlled trials
Source: Front Endocrinol (Lausanne). 2022 Jul 22;13:917692. doi: 10.3389/fendo.2022.917692 (PMC9355558; doi:10.3389/fendo.2022.917692)
Supplement: Supplementary file 1 [file DataSheet_1.docx]

**Supplementary Appendix**

**PubMed**

**#1** Search “flavonoid” [MeSH Terms]

**#2** Search “insulin resistance” [MeSH Terms]

**#3** Search (flavonoid* OR flavonol OR flavone OR flavanone OR flavan-3-ol OR anthocyanidin OR isoflavone OR quercetin OR kaempferol OR myricetin OR isorhamnetin OR luteolin OR apigenin OR hesperetin OR naringenin OR catechin OR epicatechin OR epigallocatechin OR epicatechin-3-gallate OR epigallocatechin-3-gallate OR cyanidin OR delphinidin OR malvidin OR pelargonidin OR petunidin OR peonidin OR genistein OR daidzein OR hespertin OR proanthocyanidin): Title/Abstract/Text Word

**#4** Search (“insulin resistance” OR “insulin sensitivity” OR “hyperinsulinemic euglycemia clamp” OR “euglycemic clamp” OR “glucose clamp” OR “glucose infusion rate” OR GIR OR “oral glucose tolerance test” OR OGTT OR “insulin tolerance test” OR ITT OR “homeostasis model assessment” OR HOMA OR HOMA-IAI OR HOMA-IR OR HOMA-IS OR HOMA-β OR “quantitative insulin sensitivity check index” OR QUICKI OR “fasting insulin resistance index” OR FIRI OR “insulinogenic index” OR IGI OR “minimal model analysis” OR “minimal model” OR “index of insulin sensitivity” OR “insulin sensitivity index” OR ISI OR “fasting insulin” OR FINS OR “fasting glucose” OR FPG): Title/Abstract/Text Word

**#5** Search (overweight OR obese OR obesity): Title/Abstract/Text Word

**#6** Search (‘randomized controlled trial’ OR RCT OR randomized OR clinical OR trial OR randomly): All Fields

**#7** Search #1 OR #3

**#8** Search #2 OR #4

**#9** Search **#5** AND #6 AND #7 AND #8

**Embase**

**#1** Search ((insulin AND resistance) OR (insulin AND sensitivity) OR (hyperinsulinemic AND euglycemia AND clamp) OR (euglycemic AND clamp) OR (glucose AND clamp) OR (glucose AND infusion AND rate) OR GIR OR (oral AND glucose AND tolerance AND test) OR OGTT OR (insulin AND tolerance AND test) OR HOMA OR (homeostasis AND model AND assessment) OR ITT OR 'HOMA-IAI' OR 'HOMA-IR' OR 'HOMA-IS' OR 'HOMA-β' OR (quantitative AND insulin AND sensitivity AND check AND index) OR quicki OR 'fasting insulin resistance index' OR FIRI OR (insulinogenic AND index) OR IGI OR (minimal AND model AND analysis) OR (minimal AND model) OR (index AND of AND insulin AND sensitivity) OR (insulin AND sensitivity AND index) OR ISI OR (fasting AND insulin) OR FINS OR (fasting AND glucose) OR FPG)

**#2** Search (flavonoid^*^ OR flavonol OR flavone OR flavanone OR flavan-3-ol OR anthocyanidin OR isoflavone OR quercetin OR kaempferol OR myricetin OR isorhamnetin OR luteolin OR apigenin OR hesperetin OR naringenin OR catechin OR epicatechin OR epigallocatechin OR epicatechin-3-gallate OR epigallocatechin-3-gallate OR cyanidin OR delphinidin OR malvidin OR pelargonidin OR petunidin OR peonidin OR genistein OR daidzein OR hespertin OR proanthocyanidin):ti,ab,kw

**#3** Search (overweight OR obese OR obesity): ti,ab,kw

**#4** Search (randomized AND controlled AND trial OR rct OR clinical OR randomized OR trial)

**#5** Search **#1** AND **#2** AND **#3** AND **#4**

**Web of Science**

**#1** Search (“insulin resistance” OR “insulin sensitivity” OR “hyperinsulinemic euglycemia clamp” OR “euglycemic clamp” OR “glucose clamp” OR “glucose infusion rate” OR GIR OR “oral glucose tolerance test” OR OGTT OR “insulin tolerance test” OR ITT OR “homeostasis model assessment” OR HOMA OR HOMA-IAI OR HOMA-IR OR HOMA-IS OR HOMA-β OR “quantitative insulin sensitivity check index” OR quicky OR “fasting insulin resistance index” OR FIRE OR “insulinogenic index” OR IGI OR “minimal model analysis” OR “minimal model” OR “index of insulin sensitivity” OR “insulin sensitivity index” OR ISI OR “fasting insulin” OR FINS OR “fasting glucose” OR FPG): Topic

**#2** Search (flavonoid OR flavonol OR flavone OR flavanone OR flavan-3-ol OR anthocyanidin OR isoflavone OR quercetin OR kaempferol OR myricetin OR isorhamnetin OR luteolin OR apigenin OR hesperetin OR naringenin OR catechin OR epicatechin OR epigallocatechin OR epicatechin-3-gallate OR epigallocatechin-3-gallate OR cyanidin OR delphinidin OR malvidin OR pelargonidin OR petunidin OR peonidin OR genistein OR daidzein OR hesperetin OR proanthocyanidin): Topic

**#3** Search (overweight OR obese OR obesity): Topic

**#4** Search (clinical OR trial OR randomised OR randomized OR random): Topic

**#5** Search (mice OR mouse OR rat OR rats): abstract

**#6** Search **#1** AND **#2** AND **#3** AND **#4** NOT **#5**

**Cochrane Library**

**#1** Search MeSH descriptor: [Flavonoids] explode all trees

**#2** Search (flavonoid* OR flavonol OR flavone OR flavanone OR flavan3ol OR anthocyanidin OR isoflavone OR quercetin OR kaempferol OR myricetin OR isorhamnetin OR luteolin OR apigenin OR hesperetin OR naringenin OR catechin OR epicatechin OR epigallocatechin OR epicatechin3gallate OR epigallocatechin3gallate OR cyanidin OR delphinidin OR malvidin OR pelargonidin OR petunidin OR peonidin OR genistein OR daidzein OR hesperetin OR proanthocyanidin): in All Text

**#3** Search MeSH descriptor: [insulin resistance] explode all trees

**#4** Search (“insulin resistance” OR “insulin sensitivity” OR “hyperinsulinemic euglycemia clamp” OR “euglycemic clamp” OR “glucose clamp” OR “glucose infusion rate” OR GIR OR “oral glucose tolerance test” OR OGTT OR “insulin tolerance test” OR ITT OR “homeostasis model assessment” OR HOMA OR HOMA-IAI OR HOMA-IR OR HOMA-IS OR HOMA-β OR “quantitative insulin sensitivity check index” OR quicky OR “fasting insulin resistance index” OR FIRE OR “insulinogenic index” OR IGI OR “minimal model analysis” OR “minimal model” OR “index of insulin sensitivity” OR “insulin sensitivity index” OR ISI OR “fasting insulin” OR FINS OR “fasting glucose” OR FPG): in All Text

**#5** Search **#1** OR **#2**

**#6** Search **#3** OR **#4**

**#7** Search (overweight OR obese OR obesity): in All Text

**#8** Search **#5** AND **#6** AND **#7**

**ClinicalTrials.gov registry**

**#1** Search (“insulin resistance” OR “insulin sensitivity” OR “hyperinsulinemic euglycemia clamp” OR “euglycemic clamp” OR “glucose clamp” OR “glucose infusion rate” OR GIR OR “oral glucose tolerance test” OR OGTT OR “insulin tolerance test” OR ITT OR “homeostasis model assessment” OR HOMA OR HOMA-IAI OR HOMA-IR OR HOMA-IS OR HOMA-β OR “quantitative insulin sensitivity check index” OR quicky OR “fasting insulin resistance index” OR FIRE OR “insulinogenic index” OR IGI OR “minimal model analysis” OR “minimal model” OR “index of insulin sensitivity” OR “insulin sensitivity index” OR ISI OR “fasting insulin” OR FINS OR “fasting glucose” OR FPG): Outcome Measure

**#2** Search (flavonoid OR flavonol OR flavone OR flavanone OR anthocyanidin OR isoflavone OR quercetin OR kaempferol OR myricetin OR isorhamnetin OR luteolin OR apigenin OR hesperetin OR naringenin OR catechin OR epicatechin OR epigallocatechin OR cyanidin OR delphinidin OR malvidin OR pelargonidin OR petunidin OR peonidin OR genistein OR daidzein OR hespertin OR proanthocyanidin): Intervention/treatment

**#3** Search (overweight OR obese OR obesity): Condition or disease

#**4** Search **#1** AND **#2** AND **#3**

**Supplementary Table 1.** The detailed compositions of interventions in each included RCT.

| **Author, year** | **Intervention** | **Composition** | **Explanation** |
| --- | --- | --- | --- |
| Bell, 2011 (15) | Glavonoid™ | Glavonoid™ is standardized to 30% licorice glabra polyphenol and 3% glabridin. | The main ingredients are glavonoid. |
| Cicero, 2019 (20) | Bergamot extract | The high‐dose group was given two boxes containing active treatment (bergamot extract (120mg flavonoids/pill)), and the low‐dose group was given one box containing active treatment. | The supplement contained bergamot extract (120mg flavonoids per pill) and a small amount of dry artichoke extract (which contributed only slightly to the final effect). |
| Martin, 2019 (28) | Tart cherry juice | Tart cherry juice contained 65 mg anthocyanins/L (15.6 mg/240 mL) and 33.6 g total phenolics/L (993.6 mg/240 mL). | The main ingredients are anthocyanins and phenolics. Anthocyanins belong to flavonoids. |
| Most, 2016 (30) | EGCG and resveratrol | EGCG 282 mg daily and resveratrol 80 mg daily | The main ingredient was EGCG which belongs to flavonoids. |
| Rangel-Huerta, 2015 (31) | High polyphenol concentration | A daily dose of 582.5 mg hesperidin, 125 mg narirutin, and 34 mg didymin. | The main ingredients were hesperidin and narirutin which belong to flavonoids. |
| Rondanelli, 2009 (32) | N-oleyl-phosphatidylethanolamine (NOPE) and EGCG | One capsule twice daily, 85 mg NOPE and 50 mg EGCG per capsule. | The promoted amelioration of insulin resistance could be attributable to EGCG. The effect of NOPE mainly includes reducing food intake and ameliorating in vivo plasma availability of EGCG. |
| Rondanelli, 2020 (33) | Cynara | Tablets containing 500 mg of artichoke extract (triple standardized to contain caffeoylquinic acids ≥ 5.0%; flavonoids ≥ 1.5%; cynaropicrin ≥ 1.0%) | The content of flavonoids is the main component and is only lower than that of caffeoylquinic acids. Cynaropicrin has little effect on glucose metabolism. |
| West, 2014 (36) | Cocoa/chocolate | 37 g daily of dark chocolate and a sugar-free cocoa beverage (total cocoa = 22 g/d, total flavanols = 814 mg daily) | The ingredient is high-flavanol cocoa, of which flavanols are the main active ingredient (814 mg daily). |
| Xue, 2016 (37) | Trans-resveratrol-hesperetin co-formulation | One capsule daily contained trans-resveratrol (90 mg) and hesperetin (120 mg) | The main ingredient is hesperetin 120 mg daily. |

**Supplementary Table 2.** Detailed information for flavonoids-containing supplements provided by a third-party manufacturer.

| Author, year | Intervention in the treatment group | The third-party manufacturer and the quantitation of flavonoids by established procedures |
| --- | --- | --- |
| Aubertin-leheudre, 2007 (13) | Isoflavone | Each capsule contained 17.5 mg of isoflavones extracted from natural soy, four capsules daily. The total dose of isoflavones was thus 70 mg/day, which corresponded to 44 mg of diadzein, 16 mg of glycitein, and 10 mg of genistein. Identical active capsules were supplied by Arkopharma Ltd. (Carros, France).  Arkopharma Ltd. is French phytotherapy and dietary supplement brand; all capsule products adhere to strict production specifications and undergo strict quality control at every stage of production and comply with ISO 22000 GMP (good manufacturing practice) for dietary supplements and pharmaceuticals. |
| Aubertin-leheudre, 2008 (14) | Isoflavone |  |
| Bell, 2011 (15) | Glavonoid™ | Glavonoid™ is standardized to 30% licorice glabra polyphenol and 3% glabridin, 300 mg daily. Glavonoid™ is a fixed-ingredient nutritional product available over-the-counter and complies with GMP standards. |
| Bogdanski, 2012 (16) | EGCG | One green tea extract capsule (Olimp Labs, Dębica, Poland) with their morning meal; the capsules contained 379 mg of green tea extract (including 208 mg of EGCG). Olimp Labs is a brand of pharmaceutical-grade sports nutrition products from Poland and comply with individual country GMP standards for dietary supplements and pharmaceuticals. |
| Brown, 2009 (17) | EGCG | 400 mg bid daily. Supplement packs were prepared and labeled by a third-party manufacturer (DHP, Wales, UK).  DHP is a famous brand of pharmaceutical-grade nutrition products from the UK and complies with individual country GMP standards for dietary supplements and pharmaceuticals. |
| Brüll, 2017 (18) | Quercetin | Three quercetin capsules per day (162 mg daily) (Allium cepa L.; Rudolf Wild GmbH & Company KG, Heidelberg/Eppelheim, Germany).  Rudolf Wild GmbH & Company KG is a famous brand of pharmaceutical-grade nutrition products from Germany and complies with individual country GMP standards for dietary supplements and pharmaceuticals. |
| Choquette, 2011 (19) | Isoflavones | Four capsules contain a 70 mg daily dose of isoflavones (Arkopharma Limited, Carros, France).  Arkopharma Limited is a famous brand of pharmaceutical-grade nutrition products from France and complies with individual country GMP standards for dietary supplements and pharmaceuticals. |
| Cicero, 2019 (20) | Bergamot extract | The high‐dose group was given two boxes containing active treatment of bergamot extract (120mg flavonoids/pill) (Colber®, patent number IT0001422673/EP3116520, kindly provided by Esserre Pharma srl, Rome, Italy).  Esserre Pharma srl is a famous brand of nutrition products from Italy and complies with the GMP standards. |
| Davison, 2008 (21) | Flavanol | 902 mg flavanols daily. The study was conducted at the Nutritional Physiology Research Centre at the University of South Australia (Adelaide, South Australia, Australia). The supplement complied with the GMP standards. |
| Dostal, 2015 (22) | EGCG | Four green tea extract capsules containing 1315 ±116 mg total catechins per day (843 ± 44 mg as EGCG). Capsules were supplied by Corban Laboratories (Eniva Nutraceutics) and complied with the GMP standards. |
| Guevara-Cruz, 2020 (23) | Genistein | The genistein capsules (50 mg/day) were made by a third-party manufacturer and approved for sale and use in the market by the relevant food, nutrition, or drug agencies of Mexico. |
| Hsu, 2008 (24) | Green tea extract | One capsule (400 mg) three times daily. The green tea extract samples, obtained from the Tea Research and Extension Station, Taiwan, were extracted from dried leaves of green tea according to the pre-set standard procedures with a certificate of analysis given. |
| Hsu, 2011 (25) | Green tea extract | One capsule three times daily; capsules contained 500 mg decaffeinated green tea extract. The green tea extract samples, obtained from the Tea Research and Extension Station, Taiwan, were extracted from dried leaves of green tea according to the pre-set standard procedures with a certificate of analysis given. |
| Khorshidi, 2018 (26) | Quercetin | 1,000 mg daily (Jarrow, USA).  Jarrow is a famous brand of pharmaceutical-grade nutrition products from the USA. All products are tested by ISO and USDA certified third-party laboratories, and all methods utilized by the laboratory strictly adhere to the cGMP/GLP guidelines established by the FDA. Product supervision is cGMP certified and regulated by the FDA. These regulations govern the manufacture, storage, labeling, and distribution of raw materials and finished products as they move through the supply chain. Manufacturers must evaluate the identity, strength, and composition of raw and finished products to ensure product accuracy and contamination-free. |
| Kirch, 2018 (27) | (–)-epicatechin | 25 mg daily.  (–)-Epicatechin (charge no. 9485), isolated from green tea extract, was purchased from PhytoLab. The certificate of analysis by HPLC with diode array detection, infrared spectroscopy, and ^1^H and ^13^C nuclear magnetic resonance spectroscopy confirmed the identity of (–)-epicatechin and purity of 100% by HPLC. The isolated (–)-epicatechin fulfilled all microbiological demands for preparations of category 3B of the European Pharmacopoeia. Organic solvents and inorganic impurities were not detectable according to headspace-gas chromatography and inductively coupled plasma mass spectrometry, respectively. Cadmium and mercury determined by inductively coupled plasma mass spectrometry were below the detection limit of 0.01 ppm; lead and arsenic were far below the threshold values of the European Pharmacopoeia. |
| Martin, 2019 (28) | Tart cherry juice | 240 mL daily.  Tart cherry juice (Coloma Frozen Foods) contained 65 mg anthocyanins/L (15.6 mg/240 mL) and 33.6 g total phenolics/L (993.6 mg/240 mL) and strictly adhere to the cGMP/GLP guidelines. |
| Mielgo-Ayuso, 2013 (29) | EGCG | 300 mg daily (TEAVIGO^TM^; DSM Nutritional Products). TEAVIGO^TM^ strictly adheres to the cGMP/GLP guidelines. |
| Most, 2016 (30) | EGCG and resveratrol | EGCG 282 daily and resveratrol 80 mg daily. The supplements were commercially available and were provided by Pure Encapsulations, Inc. All of the capsules were manufactured, tested, and checked following the standards of the European Union’s Good Manufacturing Practices requirements. |
| Rangel-Huerta, 2015 (31) | High polyphenol concentration | A daily dose of 582.5 mg hesperidin, 125 mg narirutin, and 34 mg didymin. High polyphenol concentration supplements were made from fresh fruit and provided by Coca Cola Europe. The high polyphenol concentration supplement (Minute Maid, Whole Press) was enriched with polyphenols that were extracted from orange albedo and pulp by a patented method and were also commercially available (745 mg in 500 mL/d). The composition of the supplement was detailed and strictly adhere to the cGMP/GLP guidelines. |
| Rondanelli, 2009 (32) | N-oleyl-phosphatidylethanolamine and EGCG | The commercially available supplement PhosphoLEAN^TM^ is a soft-gel capsule containing 85 mg NOPE extracted from soya lecithin and 121 mg of a dry green tea extract standardized at 50 mg EGCG; the capsules were manufactured by GELFIPHARMA Lodi (Milan, Italy) on behalf of CHEMI Cinisello Balsamo (Milan, Italy) and strictly adhere to the cGMP/GLP guidelines. |
| Rondanelli, 2020 (33) | Cynara | 500 mg bid daily. Tablets containing 500 mg of artichoke extract (triple standardized to contain caffeoylquinic acids ≥ 5.0%; flavonoids ≥ 1.5%; cynaropicrin ≥ 1.0%, by HPLC) were provided by Indena SpA (Milan, Italy).  Milan is a famous brand of nutrition products from Italy and complies with the cGMP standards. |
| Salden, 2016 (34) | Hesperidin 2S | Hesperidin 2S (450 mg supplied as 500 mg Cordiart) was extracted from the Citrus sinesis peel, which contained both the S and R enantiomers in the natural 4:1 S:R ratio of hesperidin. The study products strictly adhere to the cGMP/GLP guidelines. |
| Stendell-Hollis, 2010 (35) | Catechin and EGCG | 960 mL of decaffeinated green tea daily. The green tea bags comprised between 550–700 mg tea solids, providing an average catechin dose of 58.91 mg bag and 32.21 mg EGCG per bag. This study used decaffeinated green tea and herbal tea product provided by Unilever, Lipton (Unilever Bestfoods Company North America, Englewood, NJ, USA) and comply with the cGMP standards. |
| West, 2014 (36) | Cocoa/chocolate | 37 g daily of dark chocolate and a sugar-free cocoa beverage (total cocoa = 22 g/d, total flavanols = 814 mg daily). The detection methods for each component were described in detail and complied with the cGMP standards. |
| Xue, 2016 (37) | Trans-resveratrol-hesperetin co-formulation | One capsule daily contained trans-resveratrol (90 mg) and hesperetin (120 mg). The production of trans-resveratrol and hesperetin complied with the cGMP standards. The dosage of the combined prescription is obtained after a complete basic experiment before further clinical trials. |
